# Comparative analysis of mitochondrial genomes in lycoperdaceae fungi reveals intron dynamics and phylogenetic relationships

**DOI:** 10.1186/s12864-025-11911-4

**Published:** 2025-08-11

**Authors:** Xianyi Wang, Guoyu Wang, Jiawei Tao, Zhongyao Guo, Guangyin Xu, Jiayu Li, Jichuan Kang, Qin Zuo, Hongmei Liu, Qirui Li

**Affiliations:** 1 State Key Laboratory of Discovery and Utilization of Functional Components in Traditional Chinese Medicine and School of Pharmaceutical Sciences, Guizhou Medical University, Guian New District, Guizhou, China; 2 The High Efficacy Application of Natural Medicinal Resources Engineering Centre of Guizhou Province (The Key Laboratory of Optimal Utilization of Natural Medicine Resources), School of Pharmaceutical Sciences, Guizhou Medical University, Guian New District, Guizhou, China; 3 Engineering Research Center of Medical Biotechnology, School of Biology and Engineering, Guizhou Medical University, Guiyang, China; 4 Engineering and Research Centre for Southwest Bio-Pharmaceutical, Resources of National Education Ministry of China, Guizhou University, Guiyang, China

**Keywords:** Lycoperdaceae, Mitogenomes, Comparative genomics analysis, Phylogeny

## Abstract

**Background:**

The fungi of the Lycoperdaceae family are characterized by their nearly spherical fruiting bodies and possess pharmacological, economic, and ecological values. As a group of highly valuable fungi, the classification of species within the Lycoperdaceae family continues to be a subject of debate. Mitochondrial genomes typically harbor considerable number of genes and genetic elements that offer a wealth of genetic information, making them widely utilized in phylogenetic studies of eukaryotes. However, mitochondrial genome data for the Lycoperdaceae family is insufficient, and investigating its structure offers valuable insights into the evolutionary relationships between puffball fungi and other Agaricales taxa.

**Results:**

In this study, we sequenced and compared the complete mitogenomes of four species of *Lycoperdon* and *Calvatia* for the first time. Comparative analysis indicated that introns and open reading frames (ORFs) influence mitochondrial genome size variation among the four species. The gene lengths and nucleotide compositions varied across the species, and synteny analysis suggested potential gene loss during Lycoperdaceae evolution. Phylogenetic relationships of 50 Agaricales and 16 Boletales species were reconstructed based on a concatenated mitochondrial gene dataset. The results elucidated the taxonomic placements of four Lycoperdaceae species within the Agaricales, confirming the monophyly of Lycoperdaceae and its nested position within Agaricaceae. With the expansion of the phylogenetic tree, the number of introns gradually decreased across the Boletales. Additionally, significant inversion and translocation events were observed in the tRNA genes of *Lycoperdon pratense*.

**Conclusion:**

This study represents the first assembly of complete mitogenomes for four species within the Lycoperdaceae family, laying the foundation for subsequent phylogenetic research based on fungal mitochondrial gene dataset. Comparative analysis revealed the contribution of gene composition and introns to the mitochondrial genome size. Their mitochondrial genomes underwent frequent intron loss or gain events and potential intron transfer in evolution.

**Supplementary Information:**

The online version contains supplementary material available at 10.1186/s12864-025-11911-4.

## Background

Mushrooms are macrofungi that are capable of forming distinct fruiting bodies. Furthermore, mushrooms are economically, medically, and ecologically important because they have a high nutritional value and contain abundant bioactive compounds [1]. Some immature Lycoperdaceae species have edible internal spores, with delightful flavors and tender textures similar to tofu [2]. Mushrooms offer benefits to human health by boosting immunity and enhancing antioxidant capacity [3]. Lycoperdaceae, one of the renowned medicinal fungi in China, is widely distributed and can be conveniently extracted. *Calvatia* spp. contain various biologically active components and exhibit hemostatic, antioxidant, antimicrobial, and hypoglycemic effects. Thus, *Calvatia* spp. are a promising medicinal resource for wound repair, offering a solid theoretical foundation for the rationale-based development and application of these species in the clinical management of trauma [4].

The family Lycoperdaceae belongs to the suborder Agaricineae of the order Agaricales and is characterized by enclosed basidiocarps. The fungal species within this family exhibit a high degree of morphological consistency in their fruiting bodies, primarily characterized by globose morphology encompassing various forms including spherical, subglobose, sessile, and pyriform configurations. However, over time, it evolves into a powdery substance characterized by brown coloration, consisting of mature basidiospores. The gleba is enveloped by a protective peridium, which is structurally divided into the exoperidium and inner peridium. The exoperidium is commonly embellished with protuberant spines and warts, which disintegrate after maturation of the fruiting body. This process exposes the underlying endoperidium, which forms an integral part of the fungal peridium [5]. The characteristic feature of the endoperidium is its regular apical openings through which a large amount of basidiospores is expelled into the surrounding environment [6]. Later, the spores undergo passive dispersion via environmental factors, such as wind, animals, and insects.

Known for its strong environmental adaptability, Lycoperdaceae exhibits a global distribution with remarkable climatic tolerance, thriving even in extreme habitats such as Arctic tundras and arid deserts [7]. This fungal family, formerly classified under the order Lycoperdales (class Gasteromycetes), encompasses diverse puffball species [7–9]. The classification of Lycoperdaceae and Geastraceae as puffball fungi reflects the historically prevalent taxonomic concept that categorized species based on the positioning of spores either inside or on the surface of the basidiocarp. However, Fischer redefined the family, dividing it into eight genera: *Myriostoma*, *Calvatia*, *Lycoperdon*, *Lycoperdopsis*, *Disciseda*, *Bovista*, *Bovistoides*, and *Mycenastrum*. Fischer transferred *Battarrea*, *Astraeus* to Tulostomataceae, and *Astraeus* and *Geastrum* to Sclerodamerataceae and Astraceae, respectively [10].

With advances in molecular biology and fungal genetics, the classification of this species has been revised to reflect evolutionary relationships based on phylogenetic support. For instance, Krüger et al. (2001) collected fungal specimens belonging to the orders Lycoperdales and Agaricales and constructed a phylogenetic tree based on the nuc-ssu-rDNA datasets. The tree revealed that puffballs nested within the Agaricales clade with a support value of 78%, whereas Lycoperdales spp. formed distinct branches [11]. Larsson and Jeppsson conducted a systematic phylogenetic study of relevant genera within European Lycoperdaceae spp. by utilizing a comprehensive dataset of ITS and LSU-rDNA sequences in conjunction with morphological characteristics. The study yielded two significant findings: (1) Lycoperdaceae formed a monophyletic group, indicating shared evolutionary origin and (2) Lycoperdaceae and Agaricaceae were closely related (result obtained through a well-supported phylogenetic tree) [12]. Maxim et al. (2023) utilized 124 raw ITS rDNA sequences to infer the phylogenetic relationships within Lycoperdaceae, focusing on *Lycoperdon* spp. [5]. Zhao et al. (2024) conducted a comprehensive phylogenetic analysis of 96 species belonging to 19 genera within Lycoperdaceae using multigene sequences (ITS, nrLSU, *rpb2*, and *tef1*-*α*). Their study provides an extensive investigation into the phylogeny of the family and revises the taxonomic relationships among the genera. Furthermore, they propose that the morphology of capillitium types and paracapillitium may serve as useful diagnostic features for distinguishing different genera within the family [13]. These studies significantly advance our understanding of the phylogenetic relationships within Lycoperdaceae and reveal their association with Agaricales, thereby elucidating the evolutionary trajectory of these fungal taxa within the broader framework of Basidiomycota. The use of the name ‘Lycoperdaceae’ is an acceptable practice as these fungi form a monophyletic group; however, Lycoperdaceae is actually nested within Agaricaceae (some mycologists advocate that Lycoperdaceae should be treated as a tribe or subfamily under Agaricaceae, rather than as an independent family-rank taxon). However, accurately determining the number of members within Lycoperdaceae remains challenging. The primary reasons are morphological similarities among certain species within this family, as well as the lack of authoritative monographs. In addition, numerous unresolved taxonomic issues regarding the taxonomic status of new species within this family, synonymy, and phylogenetic relationships between its species and other Agaricales species render this task even more complex. Common gene fragment analysis methods, such as those based on ITS, LSU, and *rpb1* sequences, provide limited information, making it difficult to precisely identify the taxonomic status of species with similar morphology. The use of mitogenomes offers advantages in addressing these challenges.

Fungal mitochondrial genomes are typically covalently closed circular double-stranded DNA. They have a molecular weight significantly smaller than that of the nuclear genome, with a size range that is intermediate between animal and plant mitochondrial genomes [14]. Mitochondria are eukaryotic organelles characterized by a closed-loop DNA structure with a double helix. They play crucial roles in ATP production, gene expression regulation, and encode protein-coding genes (PCGs) [15]. Fungal mitochondrial genomes possess several distinctive features, including high and variable copy numbers, different genetic codes, self-splicing introns, high A/T content, gene deletions, and varying mutation rates and evolutionary rates, which differ across host organisms [16–18]. These characteristics contribute significantly to the evolution of mitochondrial genomes and the evolution of the organisms themselves, making them widely used in phylogenetic studies [19]. The primary objective of obtaining the complete mitogenome of fungi was to explore the relationship between fungal mitochondrial sequence mutations and fungal characteristics. Remarkably, the mitogenomes of fungi exhibited significant variations in terms of gene arrangement, repetitive sequences, gene number, and intron types, even among closely related species [20]. Li et al. (2023) comprehensively studied the mitogenomes of five Nidulariaceae species. Comparative analyses of gene content, gene length, tRNA, and codon usage revealed convergence within Nidulariaceae and heterogeneity within Agaricales [21].

To elucidate the systematic relationships within Lycoperdaceae and unravel the fundamental structure of its mitogenome, we performed comprehensive sequencing. In this study, we assembled and annotated the mitogenomes of four Lycoperdaceae species. Using the information obtained from genome annotation, we analyzed the gene content, tRNA gene structure, and codon usage of fungal mitogenomes. We comparatively analyzed the mitogenomes of the four species and reconstructed their phylogenetic relationships using a dataset comprising mitogenomes from 50 Agaricales and 16 Boletales species. This analysis yielded novel insights into the genetic features of the Lycoperdaceae mitogenomes.

## Materials and methods

### Specimen collection, DNA extraction, and species identification

All Lycoperdaceae species were collected from mixed or coniferous forests in Guizhou. Details of specimen collection are shown in Table S1. They were first preliminarily identified by sporocarp size, spore morphology and surface ornamentation. DNA samples were extracted from the basidiocarp of the four specimens using a fungal genomic DNA extraction kit (Solarbio, Beijing, China) according to the manufacturer’s instructions. The absorbance and concentration of the extracted DNA were determined using a NanoDrop One miniature UV–Vis spectrophotometer. After extraction, DNA was stored at − 20 °C until use. Whole-genome sequencing data for these species were obtained by NGS (Illumina HiSeq 4000 and 4 Gb raw data; Berry Genomic, Beijing, China). Dried specimens after DNA extraction were placed in specimen preservation bags containing silica gel desiccant and subsequently stored in a climate-controlled drying cabinet to ensure long-term morphological and molecular integrity. Molecular fragments were assembled using Geneious Prime v 2023.2.1 using closely related species as reference sequences. Subsequently, the sequences were aligned in the NCBI database and combined with Geneious Prime v 2023.2.1 for accurate identification by combining the results of morphological identification and molecular alignment.

### Mitogenomes assembly and annotation

The mitochondrial genome sequence was assembled de novo using approximately ten 150-bp short sequences extracted from *Amanita phalloides* (MW_ 436401) with Geneious Prime v 2023.2.1 [22]. The PCGs, open reading frames, introns, tRNA and rRNA genes of the mitogenomess were preliminarily annotated using MITOS and Mfannot according to genetic codon 4 (mold protozoan mitochondrial) [23, 24]. Additional manual proofreading is required to ensure the accuracy of the annotation results. Predicting PCGs based on genetic codon 4 using the ORF Finder tool in Geneious Prime v 2023.2.1. The results of the preliminary annotation were compared with published mitochondrial PCGs of homologous species to determine the precise start and stop codons, therefore completing the accurate annotation of PCGs for each species. The annotated rRNA and tRNA genes were further validated using RNAweasel v 5.2 and tRNAScan v 2.0.12, respectively [25, 26]. OGDraw v 1.2 was used to construct a physical map of the mitotic genome [27].

### Data analysis

The basic composition of the mitogenome was analyzed using Geneious Prime version 2023.2.1. Strand asymmetries of Lycoperdaceae mitogenomes were calculated using the following formulas: AT skew = (A − T)/(A + T), GC skew = (G − C)/(G + C). Codon usage was analyzed using MEGA 7.0 [28]. To determine the presence of interspersed repeats and intragenomic duplications of large fragments within the mitogenomes of the four Lycoperdaceae species, a blastn search was conducted on each mitogenome using a stringent threshold (*E* value < 10^−10^) [29]. Tandem repeats in the mitogenomes were determined using Tandem Repeats Finder v 4.0.9 with default parameters [30]. Forward, reverse, complemented, and reverse complemented repeats were analyzed using REPuter [31]. Mauve was employed to evaluate the collinearity of the entire genome of the four Lycoperdaceae species, starting from *cox1* [32]. Synonymous substitution rate (Ks) and nonsynonymous substitution rate (Ka) were calculated using DnaSP v 5.10.01. The Ka/Ks ratio was subsequently computed to explore evolutionary pressure [33]. This ratio distinguishes between three evolutionary scenarios: purifying selection (Ka/Ks < 1), neutral evolution (Ka/Ks = 1), and positive selection (Ka/Ks > 1), reflecting different types of evolutionary pressures that shape genetic variation. The Kimura-2-parameter (K2P) substitution model in MEGA 7.0 was used to compute average genetic distances across the 15 core PCGs within the four Lycoperdaceae species [28]. Based on insertion sites and using the *cox1* and *cob* genes of *Ganoderma calidophilum* as a reference [34], researchers classified the introns within the *cox1* and *cob* genes across ten mitochondrial genomes into distinct positional classes (Pcls). Detailed species information is provided in Table S2.

### Phylogenetic analysis

To investigate the evolutionary connections of Lycoperdaceae with other puffball fungi, we rebuilt the phylogenetic tree comprising 50 Agaricales and 16 Boletales species. The complete mitogenomes of two Gomphales species, *Ramaria rubella* (NC_068232) and *Clavulina* sp. (MT_649302), were utilised as outgroups [35, 36]. Each PCG and rRNA sequence was analysed using the Translator X online platform and the G-INS-i algorithm of MAFFT v 7.0, respectively [37, 38]. Homologous sites that were poorly aligned were eliminated using trimAI with default parameters [39]. The processed sequences were ultimately assembled into three consolidated datasets using PhyloSuite v1.2.2: PCG12 (first and second codon positions of 15 PCGs), PCG12R (first and second codon positions of 15 PCGs and two rRNAs), and AA (amino acid sequences of the 15 PCGs) [40].

The construction of phylogenetic trees may receive the influence of dataset heterogeneity, in order to get better phylogenetic results we detected the sequence composition heterogeneity of the three datasets (PCG12, PCG12R, and AA) using AliGROOVE software [41]. The three datasets were subjected to a partition homogeneity test to assess potential differences in phylogenetic signals across different genes. During the tree construction process, the best-fit evolutionary models were determined using the PartitionFinder tool within PhyloSuite v1.2.2 software, with the LG + I + G + F model applied to the AA dataset and the GTR + I + G model applied to the other datasets [40]. Phylogenetic trees were constructed using the Bayesian inference (BI) and maximum likelihood (ML) methods with MrBayes (v 3.2.7) and IQ-TREE (v 1.6.12), respectively [42, 43]. The phylogenetic trees were visualized using Figtree (v 1.4.2) [44]. For each analysis, two independent runs were conducted with four simultaneous Markov chains (one cold and four incrementally heated at T = 0.2) for 1 million generations. Sampling was performed every 1,000 generations. Once the average standard deviation of split frequencies dropped below 0.01, the initial 25% of samples were discarded as burn-in. The remaining trees were used to generate a consensus tree and calculate posterior probabilities.

## Results

### General features of Lycoperdaceae mitogenomes

**Fig. 1:**
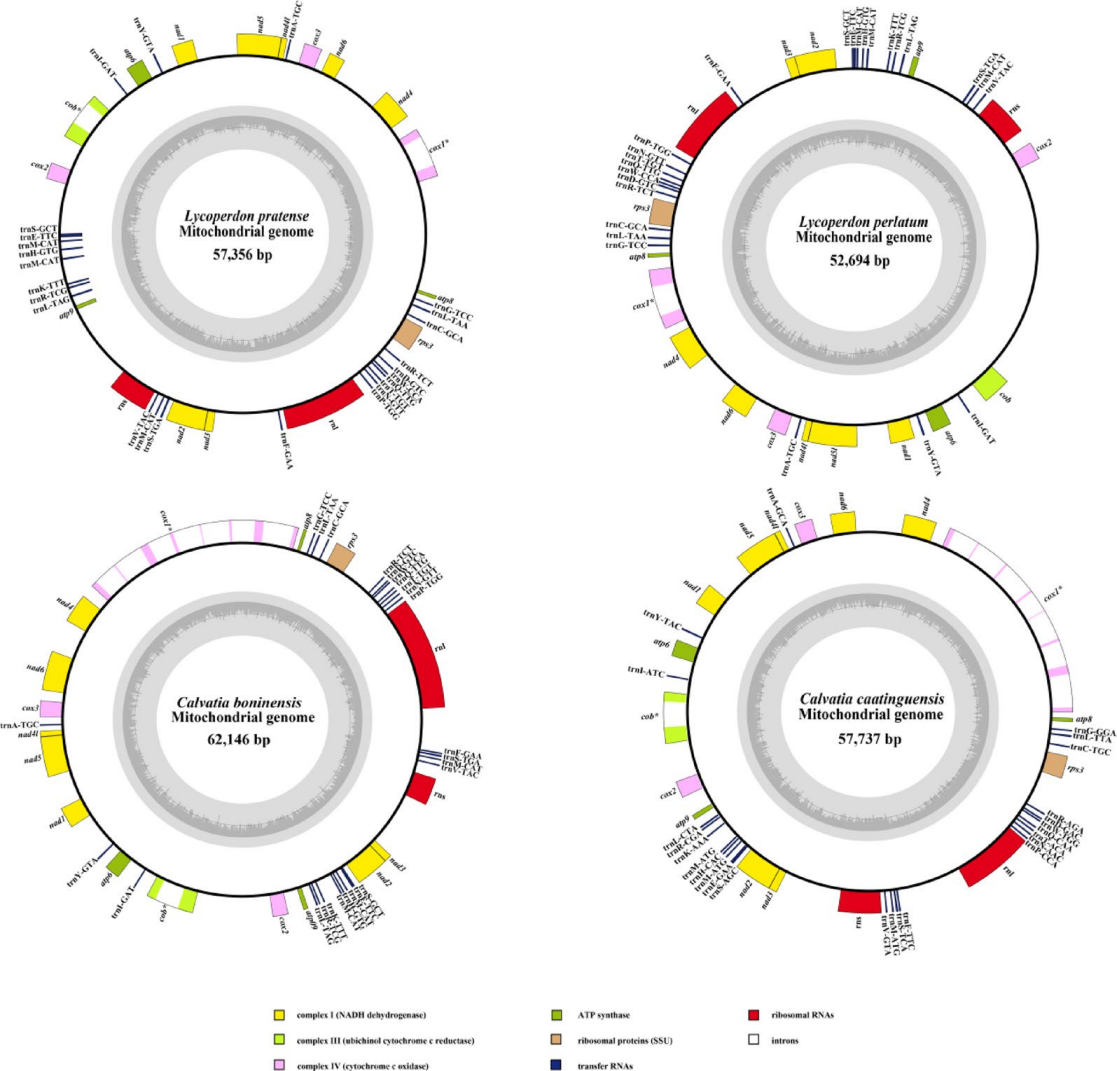
Circular maps of the four Lycoperdaceae mitogenomes. Genes are represented by different colored blocks. Colored blocks outside each ring indicate that the genes are on the direct strand, whereas colored blocks within the ring indicate that the genes are on the reverse strand

**Fig. 2:**
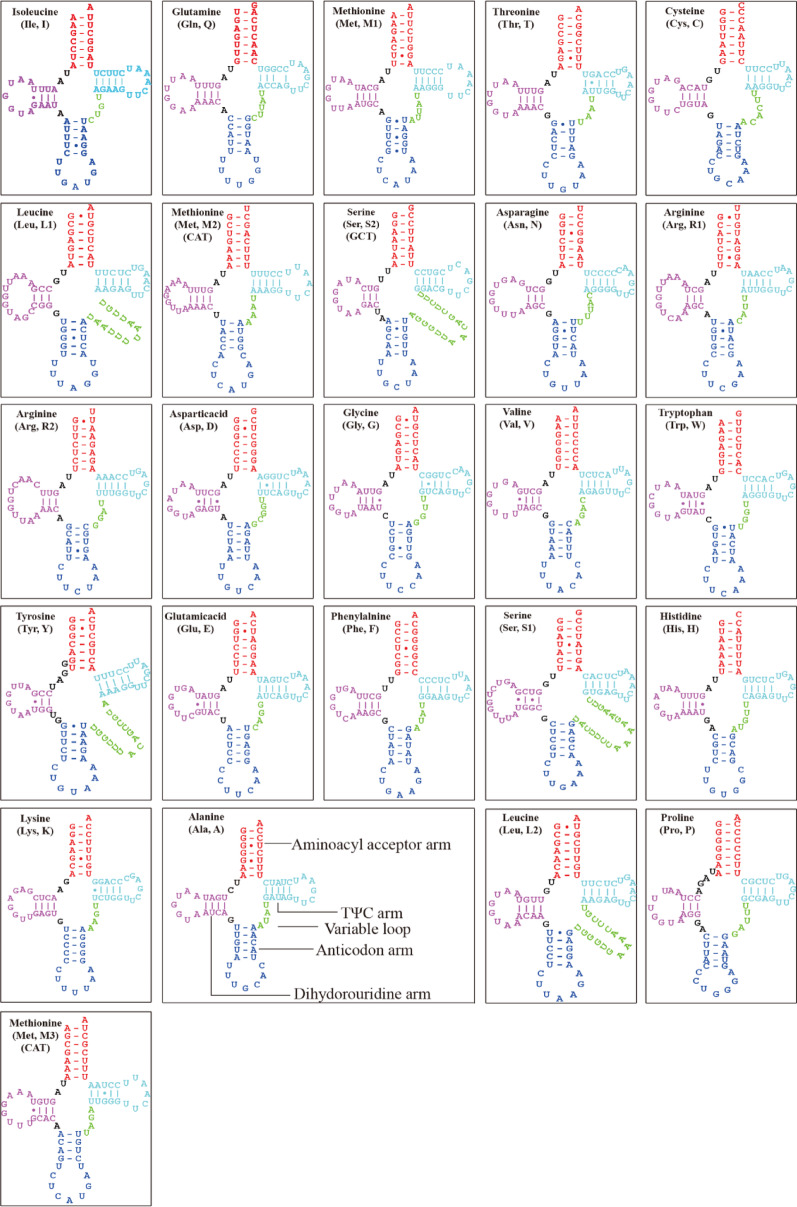
Predicted secondary structures of the 25 tRNAs of the *Lycoperdon perlatum* mitogenome

We obtained four complete mitogenomes from Lycoperdaceae. These ranged from 52,694 bp in *L*. *perlatum* to 62,146 bp in *C*. *boninensis* (*L*. *perlatum*: 52,694 bp, *L*. *pratense*: 57,356 bp, *C*. *caatinguensis*: 57,737 bp, and *C*. *boninensis*: 62,146 bp). The four newly sequenced mitogenomes contained 15 PCGs, 2 rRNA genes, and 25 tRNA genes, which encoded tRNAs for the 20 standard (Fig. 1). All four mitogenomes of Lycoperdaceae contained 15 core PCGs, including *atp6*, *atp8*, *atp9*, *cob*, *rps3*, *cox1*, *cox2*, *cox3*, *nad1*, *nad2*, *nad3*, *nad4*, *nad4L*, *nad5*, and *nad6*. The introns of these four species are distributed in *cox1* and *cob*. The mitogenomes of *Calvatia* and *Lycoperdon* spp. contained eight and two introns, respectively. The four mitogenomes contained two rRNA genes: small subunit rRNA (*rns*) and large subunit rRNA (*rnl*). The length of *rns* was 1381–1968 bp, whereas that of *rnl* was 3129–5700 bp. The possible factors contributing to the size variation in rRNA may encompass the following aspects: Mitochondrial genomes of various fungus species may experience unique evolutionary selection forces. The preservation of rRNA secondary structure frequently surpasses that of the primary sequence. Some species may preserve functional integrity after gene contraction via structural optimisation [45, 46]. These mitogenomes contained 25 tRNA genes, which are predicted to possess the typical cloverleaf secondary structure (Figs. 2, S1–S3). Simultaneously, 47–48 G–U mismatches were detected. Each mitogenome contained three tRNA genes with the same anticodons for methionine and two tRNAs with different anticodons for leucine, arginine, and serine. Most tRNA genes were located between *rnl* and *rps3* as well as between *atp9* and *nad2*, whereas the remaining ones were dispersed throughout the genome. The tRNA genes were 1,867–1,869 bp long, accounting for 3.23%–3.54% of the total length of the mitogenome. The tRNAs were 71–88 bp long, mainly due to the different sizes of the extra arms.

**Fig. 3:**
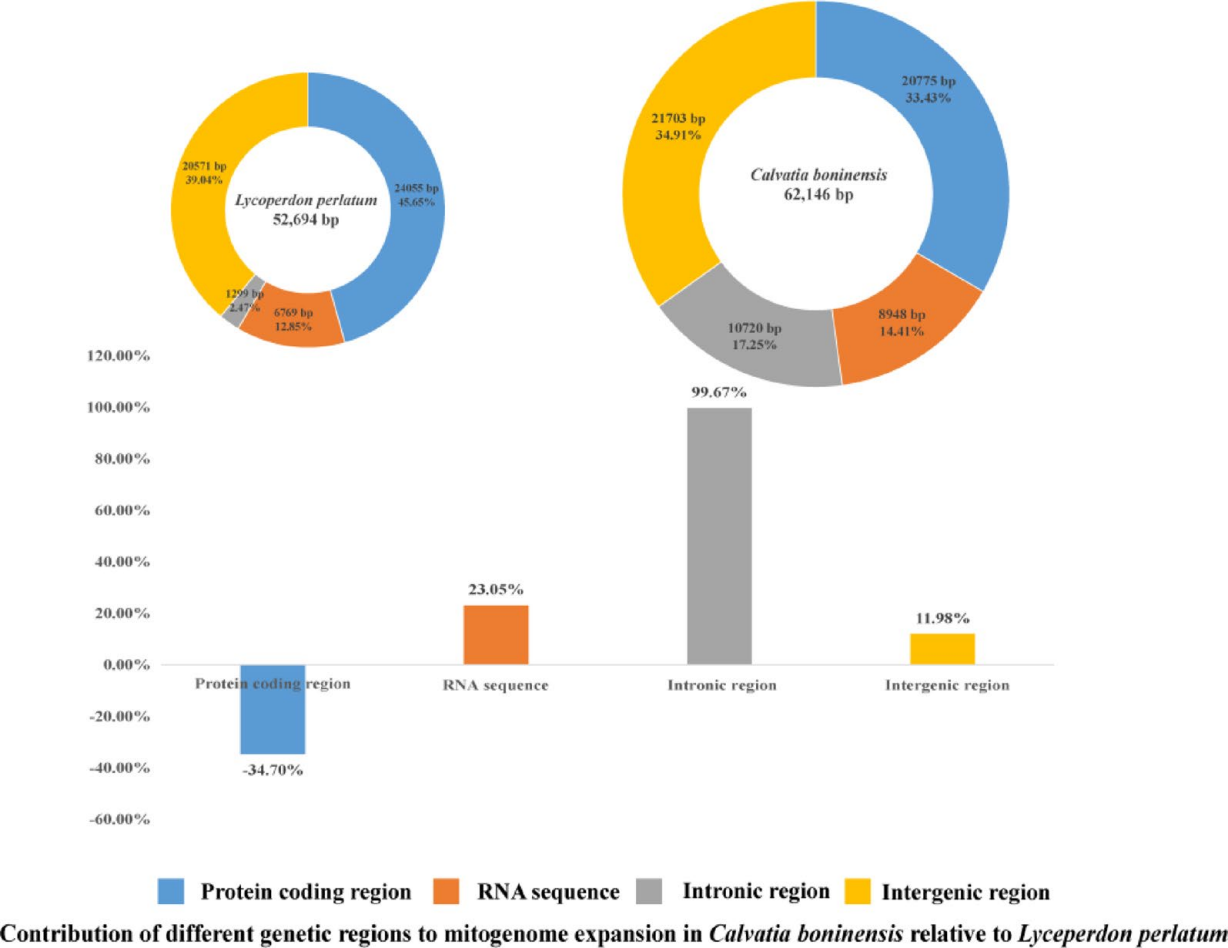
The protein-coding, intronic, intergenic, and RNA gene region proportions of the entire mitogenomes of the two Lycoperdaceae species. The bottom panel shows the contribution of different gene regions to the expansion of the *Calvatia boninensis* mitogenome

The protein-coding region accounted for the largest proportion of the *L*. *perlatum* mitogenome, totaling 45.65%. Intergenic regions occupied the largest proportion of the *C*. *boninensis* mitogenome, accounting for 34.91% (Fig. 3). The RNA coding regions (tRNA and rRNA) accounted for 12.42%–14.41% of the four mitogenomes. The mitogenome of *C*. *boninensis* was 9,452 bp larger than that of *L*. *perlatum*. Intronic regions made the greatest contribution to the expansion of the mitogenome in *C*. *boninensis*, accounting for 99.67% of the size difference (Fig. 3). In the mitogenome length expansion of *C*. *boninensis*, the protein-coding regions contributed -34.70%, whereas the intergenic regions contributed 12.01%. These results indicated that the expansion of the *C*. *boninensis* mitogenome was primarily due to the increase of intronic regions. The remaining images comparing the mitogenomes length composition between two genera are shown in Figs. S4–S6.

### Codon usage analysis

**Fig. 4:**
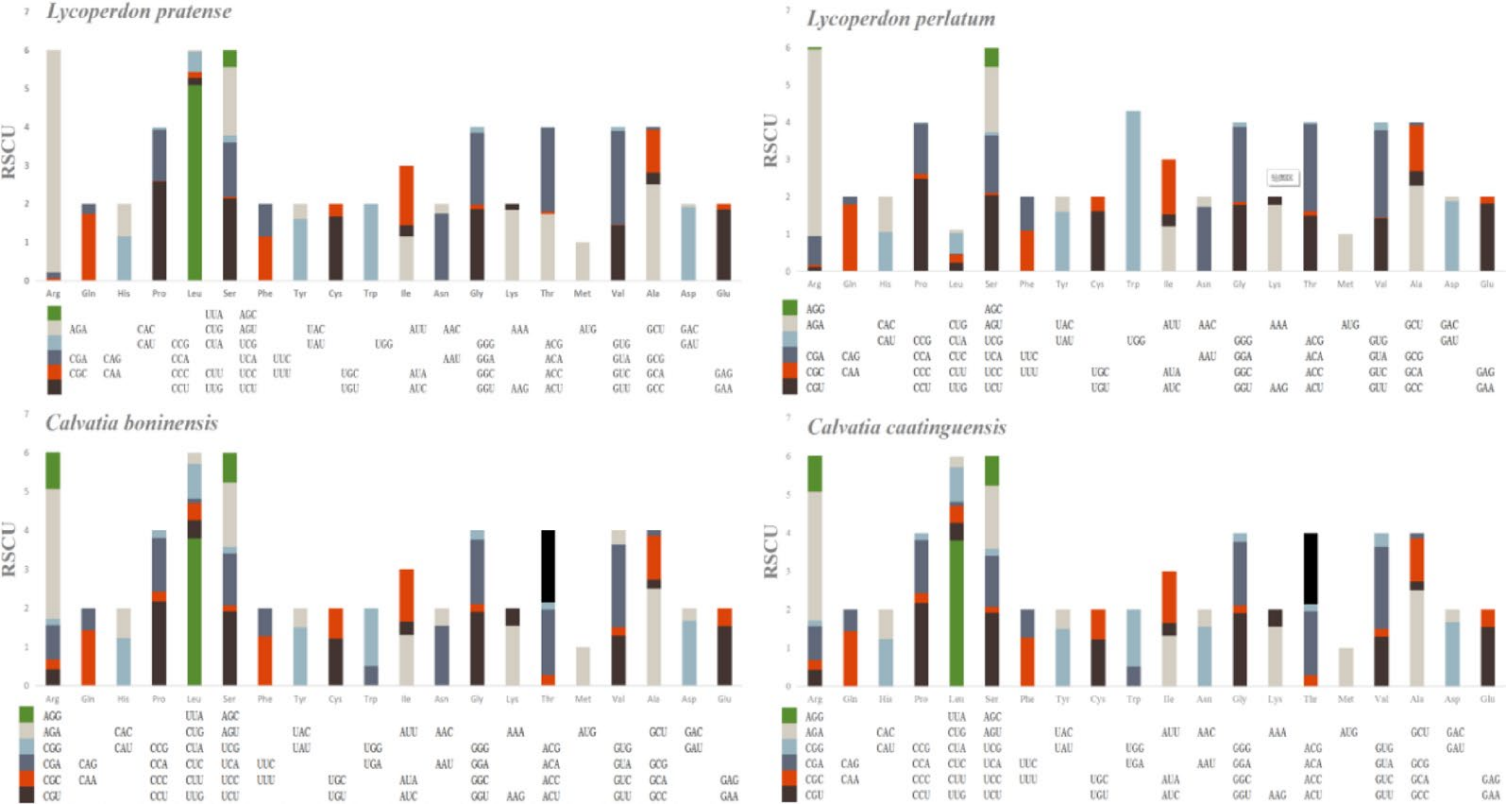
Codon usage in the four Lycoperdaceae mitogenomes. Codon families are indicated below the *x*-axis. Frequency of codon usage is plotted on the *y*-axis

ATG was the predominant start codon utilized by most core PCGs across the four mitogenomes of Lycoperdaceae. However, in the *C*. *boninensis* mitogenome, *nad1* used TTA, *atp6* used ATT, and *atp9* used TTA. In the mitogenome of *C*. *caatinguensis*, *nad1*, *nad2*, *nad6*, *atp8*, and *atp9* utilized ATA, whereas *nad4* utilized TTA. In the mitogenome of *L*. *perlatum*, *nad1*, *atp8*, and *atp9* used ATT, *nad3*, *nad4*, and *nad2* utilized TTG, and *cob* used TTA. In the mitogenome of *L*. *pratense*, *nad1* employed TTA, whereas *atp6* utilized ATA. TAA, followed by TAG, was the most commonly used stop codon in the core PCGs of the mitogenomes of the four Lycoperdaceae species (Fig. 4; Table S3).

Codon usage analysis indicated that CCT (for proline, Pro), AGA (for arginine, Arg), GTA (for valine, Val), TCT (for serine, Ser), GCT (for alanine, Ala), and TTA (for leucine, Leu) were the most frequently used codons in the four Lycoperdaceae mitogenomes. The usage of codons indicated that the higher occurrence of AT content results in the relatively higher AT content in four mitogenomes (averaging 65.63%).

### Repetitive sequence analysis

The mitogenomes of the four species were self-compared using blastn search. In total, 29, 15, 21, and 43 repeat sequences were identified in the mitogenomes of *C*. *boninensis*, *C*. *caatinguensis*,* L*. *perlatum*, and *L*. *pratense*, respectively (Table S4). The length of the repeats was 35–179 bp, with of 80.92%–100% pair-wise nucleotide similarity. The largest repeat sequence was observed in the mitogenome of *L*. *pratense*. The largest repeat region in the mitogenome was 141, 141, and 136 bp for *C*. *boninensis*, *C*. *caatinguensis*, and *L*. *perlatum*, respectively. The mitogenome of *L*. *pratense* contained the largest proportion of repeat regions, accounting for 4.63% of the mitogenome, followed by that of *C*. *boninensis* (3.14%), *C*. *caatinguensis* (2.03%), and *L*. *perlatum* (2.84%). In total, nine, nine, seven, and five tandem repeats were detected in the mitogenomes of *C*. *boninensis*, *C*. *caatinguensis*, *L*. *perlatum*, *L*. *pratense*, respectively (Table S5). The mitogenome of *L*. *perlatum* had the longest tandem repeat sequence, comprising 32 bp and encompassing 2.2 copies. The mitogenome of* L*. *perlatum* had the highest copy number of tandem repeat sequences at 5.5. Tandem repeat sequences accounted for 0.61%, 0.68%, 0.72%, and 0.30% of the mitogenomes of *C*. *boninensis*, *C*. *caatinguensis*, *L*. *perlatum*, and *L*. *pratense*, respectively. A total of 24 forward, 18 palindromic, and 2 reverse repeats were identified by REPuter in the mitogenome of *C*. *boninensis*, accounting for 4.83% of the mitogenome (Table S6). Repeat sequences accounted for 4.55%, 5.08%, and 5.78% of the *C*. *caatinguensis*, *L*. *perlatum*, and *L*. *pratense* mitogenomes, respectively.

### Evolution and variation of core PCGs

**Fig. 5:**
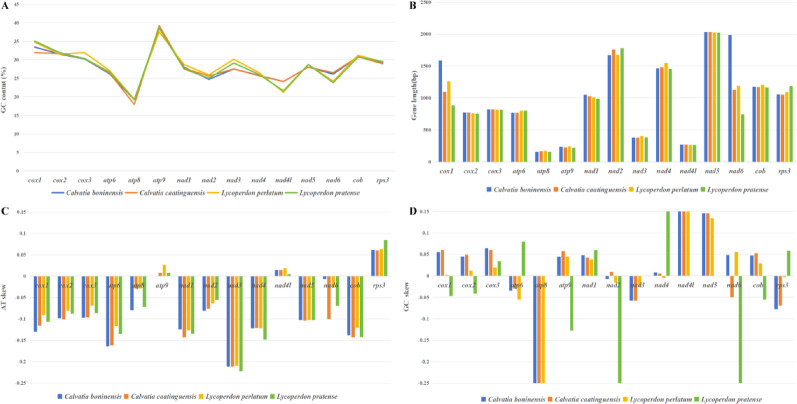
Sequence information of core protein-coding genes in the four Lycoperdaceae species. **A** GC content; **B** Gene length; **C** AT skew; **D** GC skew

**Fig. 6:**
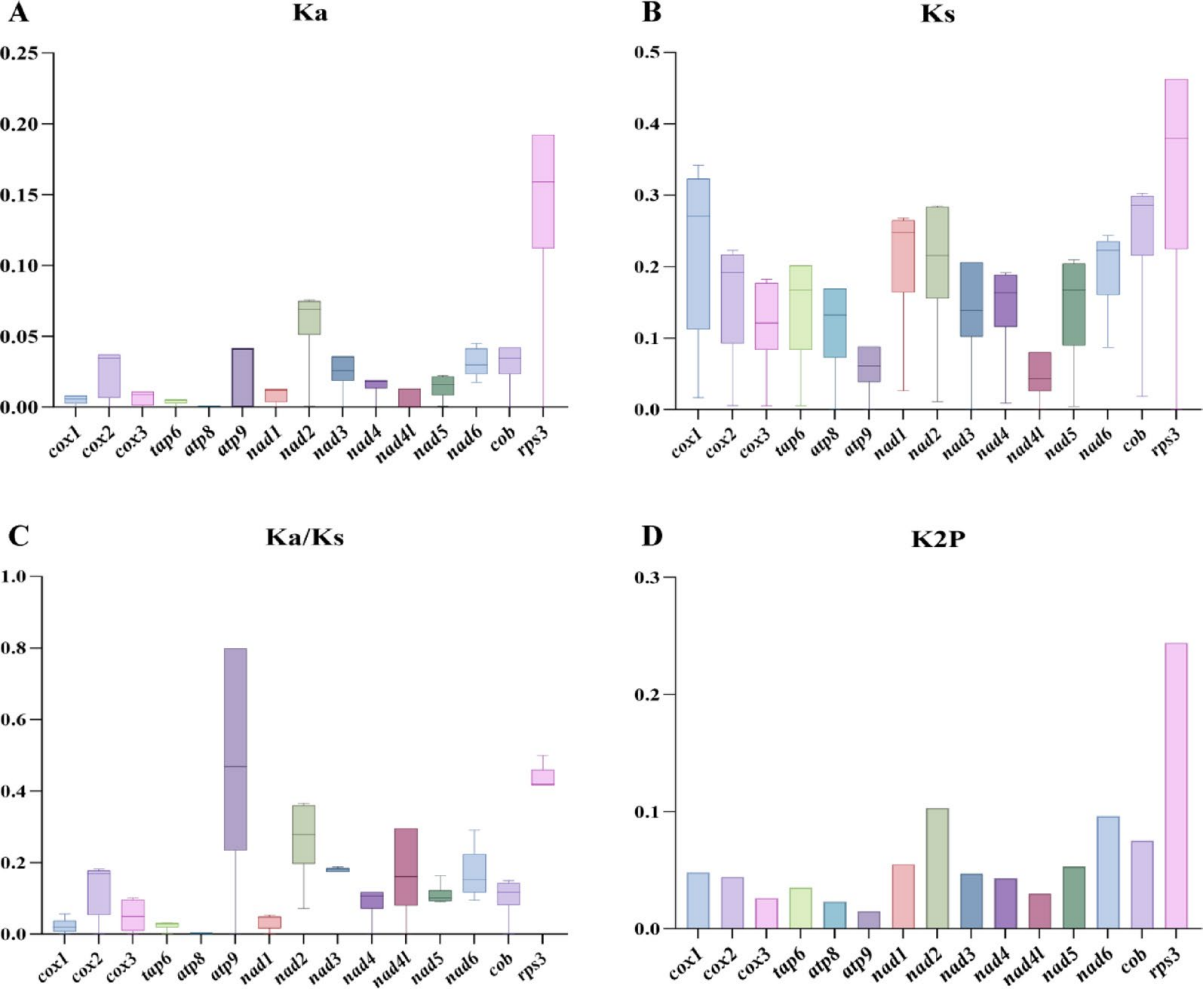
Genetic analysis of the 15 core protein-coding genes in the four Lycoperdaceae species. **A** Ka—the number of nonsynonymous substitutions per nonsynonymous site; **B** Ks—the number of synonymous substitutions per synonymous site; **C** Ka/Ks ratio—the ratio of nonsynonymous to synonymous substitution rates; **D** K2P (Kimura 2-parameter) genetic distance

The 15 core PCGs of the four mitogenomes exhibit length variation (Fig. 5). Length variation was largest for *nad6* among the 15 core PCGs detected (up to 1242 bp). Average GC content was highest for *atp9* (38.43%), followed by *cox1* (33.75%). GC content was lowest for *atp8*, at an average of 18.90%. Across the four Lycoperdaceae species, 13 of 15 core PCGs contained negative AT skews, and only *atp9* and *nad4L* contained positive AT skews, indicating that most core PCGs tend to evolve in the direction of T-rich rather than A-rich in the leading strand of core PCGs. The GC skews of core PCGs across Lycoperdaceae species varied, indicating frequent G or C mutations. Of the 15 core PCGs identified across the four Lycoperdaceae species, *rps3* exhibited the highest average K2P genetic distance, followed by *nad3*, indicating significant evolutionary differentiation among these genes (Fig. 6). Across the four Lycoperdaceae species, *atp9* had the smallest average K2P genetic distance, indicating that *atp9* is highly conserved. Of the 15 core PCGs, *rps3* had the largest Ka value, followed by *nad2* and *nad6*, and *atp8* and *atp9* had the smallest Ka value. The value of Ks was largest for *rps3* and smallest for *nad2,* among the 15 core PCGs. All 15 core PCGs Ka/Ks ratio was < 1, indicating purifying selection.

### Gene rearrangement, collinearity analysis, and heterogeneity test

**Fig. 7:**
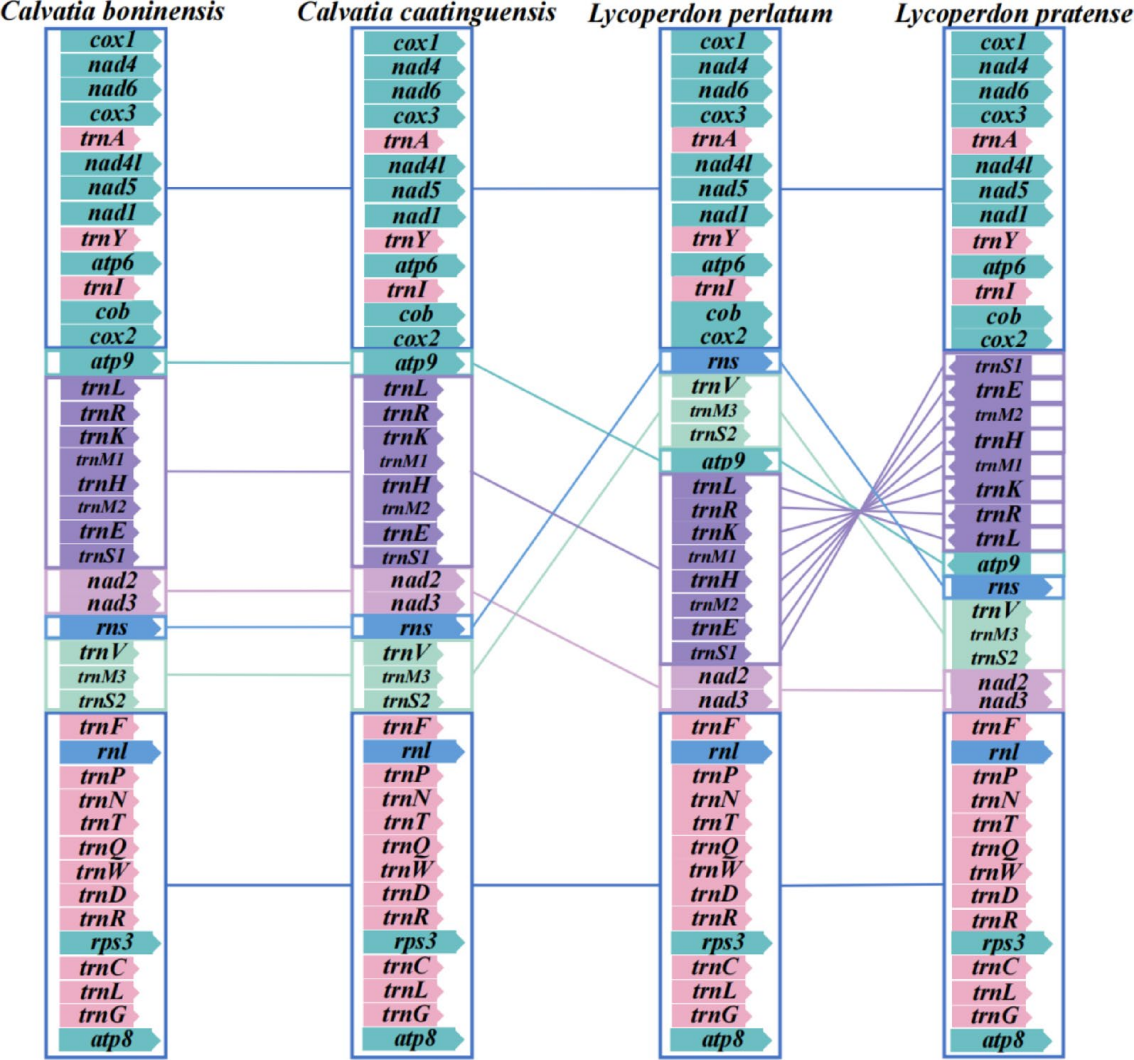
Mitochondrial gene arrangement analyses of the four Lycoperdaceae species. Genes with unchanged order are represented by blocks of the same color, genes with changed order are represented by blocks of another color, and different types of genes are distinguished by blocks with significant color differences

We compared the mitochondrial gene rearrangements of the four Lycoperdaceae species, including core PCGs, rRNA genes, and tRNA genes (Fig. 7). Most core PCGs are highly conserved across the four mitogenomes. Only *atp9* had gene replacement in the mitogenomes of two *Lycoperdon* spp. In the mitogenomes of *Lycoperdon* spp., 11 tRNAs, including *trnS1*, *trnS2*, *trnE*, *trnM1*, *trnM2*, *trnM3*, *trnH*, *trnK*, *trnR*, *trnL*, and *trnV*, underwent translocation. Additionally, in *L. pratense*, eight tRNA genes (*trnS1*, *trnE*, *trnM1*, *trnM2*, *trnH*, *trnK*, *trnR*, and *trnL*) exhibit altered transcriptional orientations alongside the *atp9* gene. For rRNA genes, only *rnl* in *L*. *perlatum* underwent translocation.

**Fig. 8:**
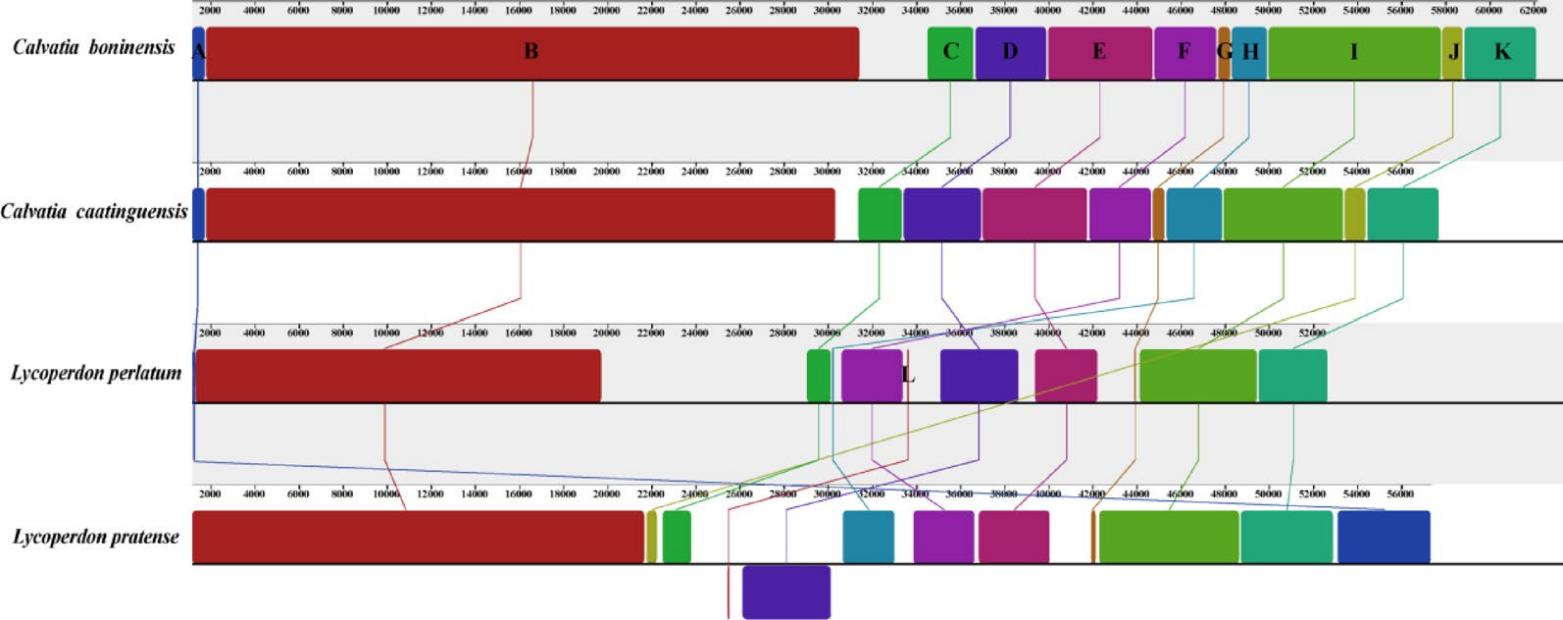
Colinearity analysis of the four Lycoperdaceae mitogenomes. Twelve homologous regions were detected across the four mitogenomes. The sizes and positions of homologous regions varied across the mitogenomes

Collinearity analysis indicated that the Lycoperdaceae mitogenome underwent gene shuffling (Fig. 8). The four Lycoperdaceae mitogenomes were divided into 12 homologous regions, with differences in size and relative position among homologous regions. Out of the 12 homologous regions in the mitochondrial genomes of the four species, 10 homologous regions are shared. Homologous region B contributed to the size of the mitogenome of *C*. *boninensis*, which was the largest among the four mitogenomes of Lycoperdaceae described in this study. There was a high degree of collinearity between *C*. *boninensis* and *C*. *caatinguensis*. During evolution, the loss of some homologous regions and the emergence of new homologous regions increased genome length.

**Fig. 9:**
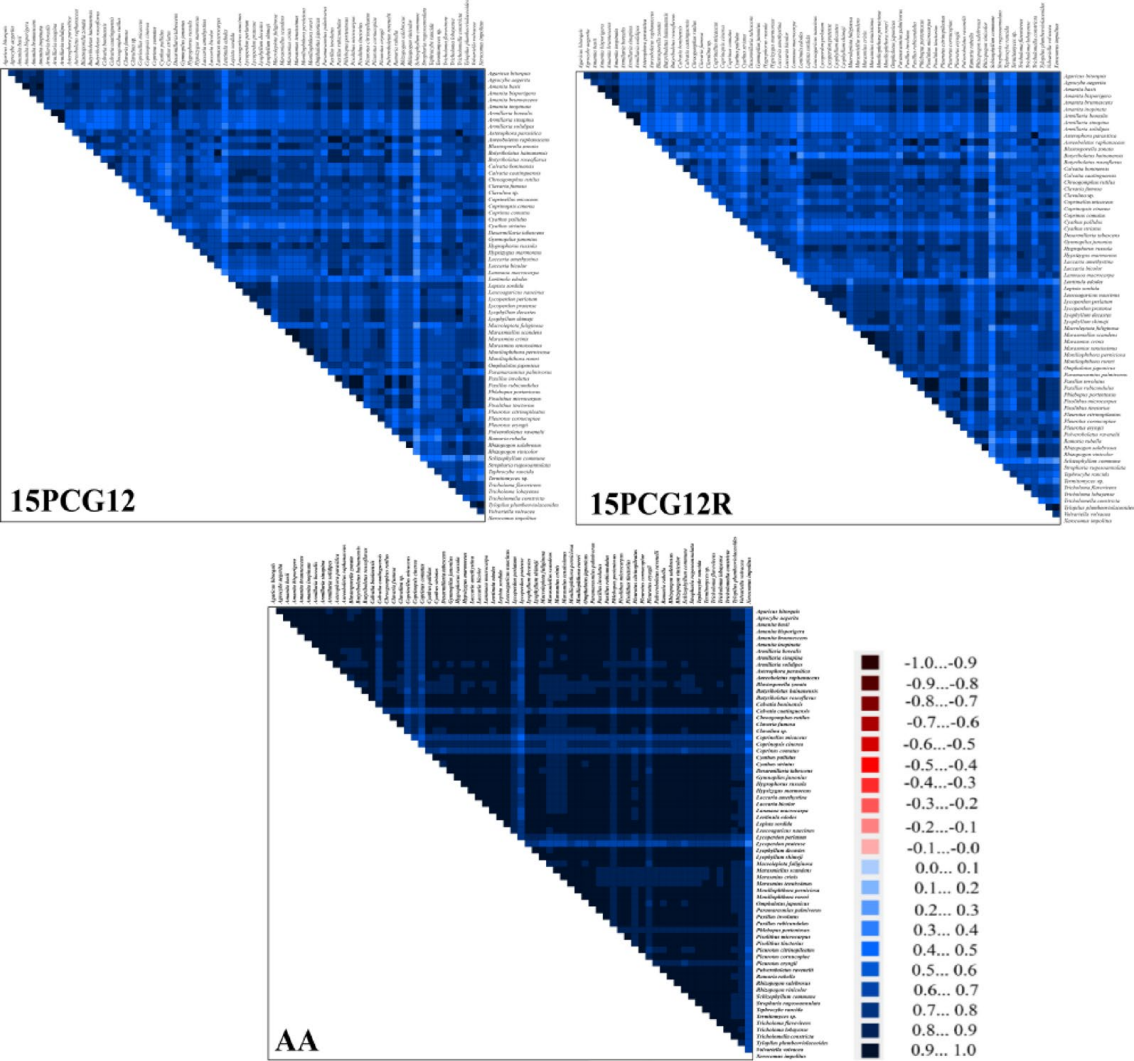
Heterogeneity analysis of the 15PCG12, 15PCG12R, and AA datasets. Heterogeneity differences between sequences are represented by color, with deep red (− 1) to deep blue (+ 1) representing a shift in heterogeneity from heavy to light, respectively

The AA dataset exhibited the lowest compositional heterogeneity among the tested datasets. The 15PCG12R dataset showed higher compositional heterogeneity than 15PCG12, with all three datasets displaying heterogeneity values significantly greater than zero. These results confirm that all three datasets meet the essential criteria for reliable phylogenetic reconstruction (Fig. 9).

### Intron dynamics in the *cox1* and *cob* genes across 10 mitogenomes and open reading frame analysis

104 introns were identified in the *cob*, *cox1*, *cox2*, *cox3*, *nad1*, *nad4*, *nad4L*, *nad5*, *rns*, and *rnl* genes of 8 Agaricales and 2 Boletales mitogenomes. Of the 104 introns, five were categorised as Group II [47], one was an unclassified group intron, and the rest were categorised as Group I. Putative open reading frames (ORFs) were identified in 83 out of 104 introns, with about 79.81% of these introns harbouring putative ORFs that encode homing endonucleases, maturases, or reverse transcriptases [48, 49]. These introns had an irregular distribution among host genes. The *cox1* and *cob* genes included the largest numbers of introns, comprising 55.77% and 16.67% of the total introns, respectively. This work demonstrated that introns preferentially insert into particular genes, including *cox1* and *cob*, a behaviour considered to be independent of host gene length [50]. In the ten mitochondrial genomes, the *atp6*, *atp8*, *atp9*, *nad2*, *nad3*, and *nad6* genes were entirely without introns. The fluctuating presence of introns in the *cox1* and *cob* genes may substantially affect the structural configuration and dimensions of mitochondrial genomes in Agaricales.

**Fig. 10:**
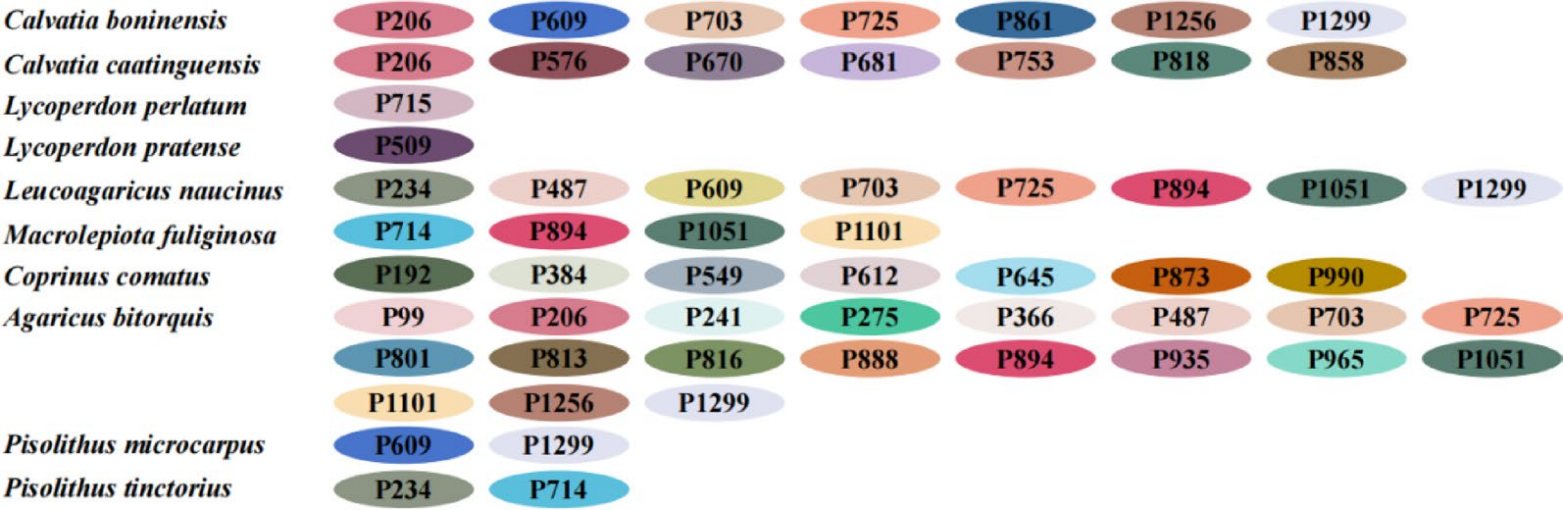
The positional class (Pcl) information of the *cox1* gene in 4 Lycoperdaceae, 4 Agaricaceae, and 2 Pisolithaceae species. Introns in *cox1* genes of the 10 mitogenomes were classified into different position classes (Pcls) using the *cox1* gene of *Ganoderma calidophilum* as the reference

**Fig. 11:**
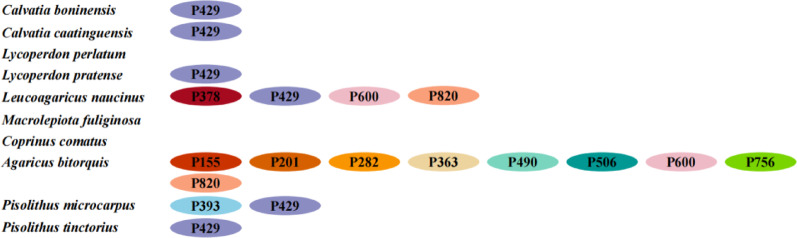
The positional class (Pcl) information of the *cob* gene in 4 Lycoperdaceae, 4 Agaricaceae, and 2 Pisolithaceae species. Introns in *cob* genes of the 10 mitogenomes were classified into different position classes (Pcls) using the *cob* gene of *Ganoderma calidophilum* as the reference

In this study, we investigated intron dynamics within Lycoperdaceae and Pisolithaceae species of puffballs, as well as between Lycoperdaceae and their closely related species in Agaricaceae. To do this, introns in the *cox1* and *cob* genes from 10 mitochondrial genomes were categorized into distinct position classes (Pcls) (Figs. 10, 11). The 58 introns in the *cox1* gene from 10 mitochondrial genomes were classified into 39 Pcls, while the 18 introns in the *cob* gene were categorized into 12 Pcls, demonstrating the remarkable diversity of intron types in the *cox1* gene within the order Agaricales. The interspecific variance in intron types and numbers suggests potential intron loss or gain events during the evolution of the order Agaricales. P206, P703, P725, P894, P1051, and P1299 are extensively disseminated Group I introns within the *cox1* gene of Agaricales mushrooms and are present in five out of the eight Agaricales fungi examined. Additionally, intron P1299 was identified in *Pisolithus microcarpus*. The introns P1101, P609, and P487 are the second most common Group I introns in the *cox1* gene, occurring in 4 of the 8 examined Agaricales mitochondrial genomes. P609 was also found in *P*. *microcarpus*. The P429 intron was identified in the *cob* gene of seven out of ten species.

We investigated the quantity and length distribution of open reading frames (ORFs) located among protein-coding genes (PCGs), tRNA, and rRNA regions in four Lycoperdaceae mitochondrial genomes. The mitochondrial genomes of *C*. *boninensis* and *C*. *caatinguensis* contained 8 and 10 ORFs, respectively. Of these, a total of three ORFs (across both species) were identified as potential plasmid-derived pseudogenes [51]. The total ORF lengths reached 6,485 bp (11.23% of the mitochondrial genome) in *C*. *boninensis* and 11,528 bp (18.55% of the mitochondrial genome) in *C*. *caatinguensis*. In *L*. *perlatum* and *L*. *pratense* mitochondrial genomes, 14 and 12 ORFs were detected respectively, with two showing characteristics of plasmid-derived pseudogenes. The cumulative ORF lengths were 13,219 bp (25.10% of the mitochondrial genome) for *L*. *perlatum* and 10,264 bp (17.90% of the mitochondrial genome) for *L*. *pratense*.

### Phylogenetic analysis

**Fig. 12:**
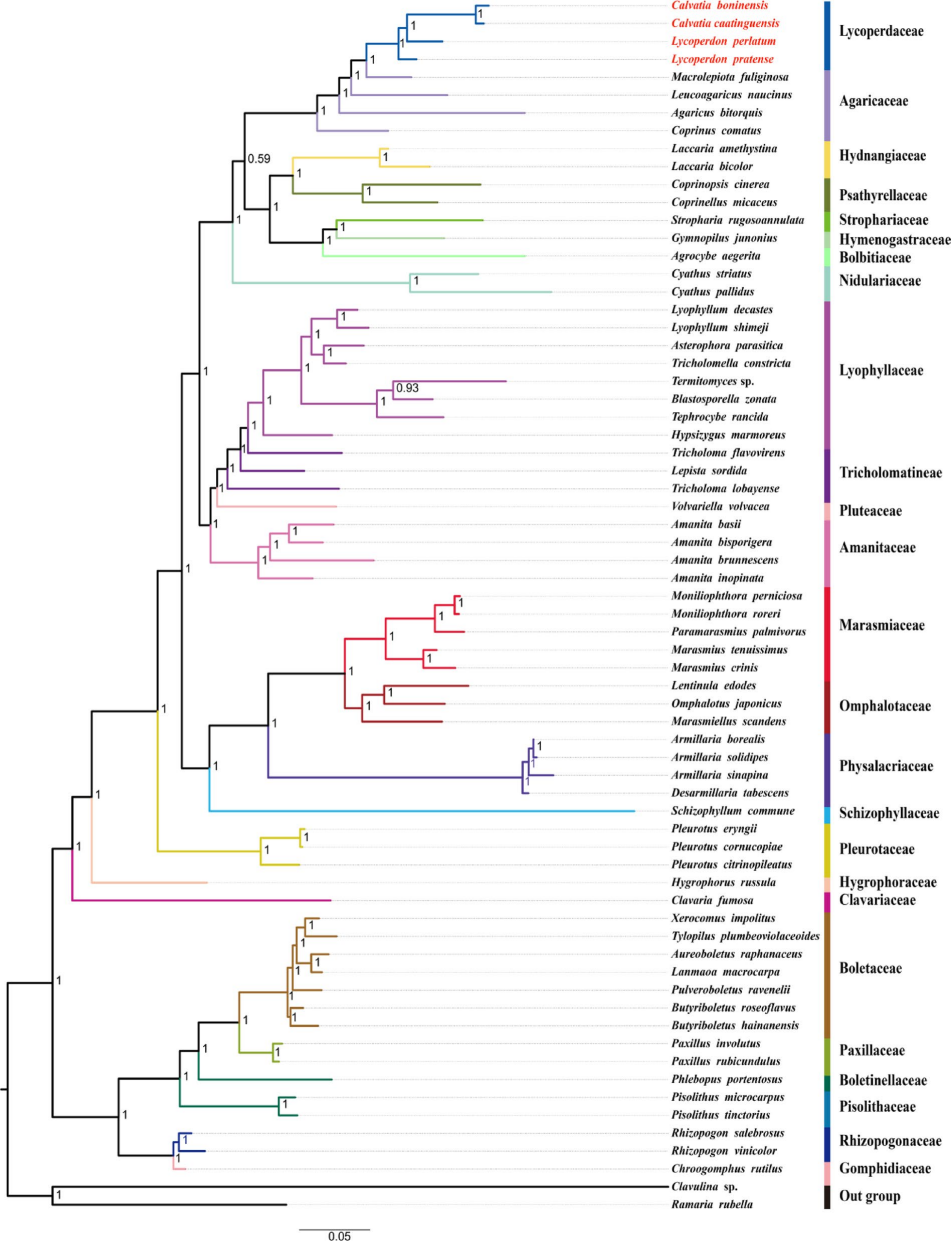
Phylogenetic reconstruction of 50 Agaricales and 16 Boletales species. The tree was inferred using Bayesian Inference (BI) under the best-fit evolutionary model GTR + I + G with the 15PCG12R dataset. Analyses were performed in MrBayes v 3.2.7

In this study, to investigate the phylogenetic relationships between the Lycoperdaceae fungi of Agaricales and other puffball-shaped fungi in Boletales, we reconstructed the phylogenetic trees of 50 Agaricales species and 16 Boletales species. To obtain additional evidence for taxonomic classification and understanding the evolutionary history of mitochondrial genomes, this study constructed phylogenetic trees of 66 species using Bayesian Inference (BI) and Maximum Likelihood (ML) based on three datasets. In total, six molecular phylogenetic trees were obtained—six topological structures (BI/ML-AA, BI/ML-15PCG12, and BI/ML-15PCG12R) (Table S7; Figs. 12, S4−S8). Support rate was higher for BI trees than for ML trees. The four Lycoperdaceae species clustered together. According to the phylogenetic tree, the 66 species could be divided into 24 major clades, corresponding to the families Hydnangiaceae, Psathyrellaceae, Strophariaceae, Hymenogastraceae, Nidulariaceae, Lycoperdaceae, Agaricaceae, Lyophyllaceae, Tricholomataceae, Amanitaceae, Pluteaceae, Marasmiaceae, Omphalotaceae, Physalacriaceae, Schizophyllaceae, Pleurotaceae, Hygrophoraceae, Boletaceae, Paxillaceae, Boletinellaceae, Pisolithaceae, Rhizopogonaceae, Gomphidiaceae, and Clavariaceae. The preliminary phylogenetic reconstruction suggested *C*. *boninensis* and *C*. *caatinguensis* form sister lineages in the current analysis. However, we acknowledge this relationship requires verification through expanded taxon sampling given that only two *Calvatia* species were included in this study. *L*. *perlatum*, as a separate branch, gathered within Lycoperdaceae and belonged to the ancestral sister species of these four Lycoperdaceae species. Lycoperdaceae showed a close affinity to Agaricaceae, whereas Pisolithaceae shared a close relationship with Boletinellaceae. These two conclusions are consistent with findings of previous studies [7, 52].

## Discussion

“Gasteroid fungi” refers to fungi that enclose their spores within the basidiocarps without exposing them when immature. They usually appear spherical or lumpy, with no visible structures, such as caps or gills. The class Gasteromycetes was established to encompass these species, given their extensive diversity, including puffballs, earthstars, stinkhorns, and bird’s nest fungi [53]. The family Lycoperdaceae, examined in this study, represents one lineage of puffball fungi. Notably, puffballs also incorporate certain taxa within Boletales (e.g., *Pisolithus*, *Astraeus*, Scleroderma), *Tulostoma* (Agaricales), and *Geastrum* (Geastrales), among others [10]. The Lycoperdaceae family examined in this study represents perhaps a taxononomically cohesive group of puffballs.

Mitogenomes are crucial for eukaryotic growth and development, oxidative stress responses, and environmental adaptation [54, 55]. Mutations in the mitogenome of eukaryotic organisms may lead to hereditary and multisystem diseases that affect animal metabolism [56]. Although fungi are one of the most species-rich groups, research on the mitogenome of Lycoperdaceae is severely lacking [57]. The complete mitogenome of Lycoperdaceae has not been reported in public databases. This is due to the complexity of fungal mitogenomes sequencing and assembly, including gene content, highly repetitive sequences, complex genome structure and genome length variations. As a result, research in this area is ongoing [58]. Compared with other eukaryotes, there is a considerable variation in mitogenome sizes among closely related fungal species [59]. Fungal mitogenomes exhibit high variability in size, primarily due to variations in introns, repetitive sequences, and intergenic regions, as well as the accumulation of horizontally transferred genes [20, 60–63]. The present study revealed that the mitogenome of *C*. *boninensis* is the largest among the four examined species in the family Lycoperdaceae. This mitogenome contains 10 ORFs interspersed within the sequences of PCGs, tRNAs, and rRNAs, totaling 11,528 bp. Additionally, eight putative intronic open reading frames were identified, with a combined length of 7,798 bp. The mitogenome of *C*. *boninensis* contains the highest numbers of introns (8) and open reading frames (18) reported in the Lycoperdaceae family. Comparative analysis revealed 5–9 types of repetitive sequences across four mitogenomes, accounting for only 0.30%-0.71% of the total genome length. These findings indicate that the quantity of open reading frames and introns constitutes the primary factor driving mitogenome size variation within Lycoperdaceae species.

Introns are mobile selfish genetic elements within fungal mitochondrial genomes, capable of inserting or being lost with relative ease [64]. Li et al. (2022) previously compared intron dynamics in the mitochondrial genomes of six Phallomycetidae species spanning three orders. Their study revealed intron mobility events among these six species; however, comparisons with more closely related families within these orders were lacking. Furthermore, Pisolithaceae and Lycoperdaceae exhibit similarities in morphology and spore dispersal mechanisms. Therefore, the present study selected four species of Lycoperdaceae, four species of the closely related Agaricaceae, and two species of Pisolithaceae for intron dynamics analysis. In this study, Agaricales exhibited substantial variation in intron quantity and type, ranging from 1 intron in *Lycoperdon perlatum* to 47 introns in *Agaricus bitorquis*. The diversity of *cox1* intron types spanned from 1 to 18 distinct categories. Notably, while intron types exhibit remarkable diversity, certain intronic elements recurrently appear across divergent species (P609, P703, P725). Notably, while intron types exhibit remarkable diversity, certain intronic elements recurrently appear across divergent species. For instance, the P234 intron was found in both *P*. *tinctorius* and *Leucoagaricus naucinus*, and the P714 intron in both *P*. *tinctorius* and *Macrolepiota fuliginosa* (order Agaricales). In contrast, the P861 intron [65–67] was not observed in these species. Introns classified within the same Pcls (position classes) groups are considered homologous, exhibiting conserved sequence architectures and functional characteristics [68]. During the evolution of the Lycoperdaceae, the homing endonucleases encoded by the P206 intron in the *cox1* gene and the P429 intron in the *cob* gene play critical roles in DNA repair and recombination. Thus, these introns have been retained through potential intron transfer events. Meanwhile, we found that Pcls of all introns in *Leucoagaricus naucinus* were detected in both closely and distantly related species, and the reasons behind this phenomenon are not understood but warrant future investigation.

The ancestors of eukaryotes acquired mitochondria from bacteria through endosymbiosis, and over the course of evolution, most mitochondrial genes were relocated to the nuclear genome [69, 70]. However, some PCGs, tRNA genes, and rRNA genes are retained in the mitogenome [71]. Most publicly available fungal mitogenomes include 15 core PCGs: *atp6*, *atp8*, *atp9*, *cob*, *cox1*, *cox2*, *cox3*, *nad1*, *nad2*, *nad3*, *nad4*, *nad4L*, *nad5*, *nad6*, and *rps3*. Studies have revealed significant differences in both the length and nucleotide composition of core PCGs not only among the mitogenomes of the four species within Lycoperdaceae, but also among other species within Agaricales [72]. The impact of core PCG mutations on fungal growth requires further investigation. Additionally, different core PCGs exhibit varying evolutionary rates and are likely subject to purifying selection. We also identified partial site mutations in the 25 tRNAs within the mitogenomes of the four Lycoperdaceae species. tRNA mutations are associated with protein synthesis efficiency and may ultimately impact the phenotype of eukaryotes [71].

The rearrangement of mitochondrial genes is associated with the increase in genetic diversity and the evolutionary differentiation of species. Mitogenome rearrangement has been studied in animals and plants, revealing the relationship between species evolution and gene rearrangement [73, 74]. However, in contrast to animals, the mitochondrial gene sequence of fungi is highly variable, even among closely-related species [75]. Mitochondrial gene rearrangements have been identified in most mitogenomes from mushroom-forming fungi (Agaricomycetes) [76]. This phenomenon could be attributed to the accumulation of repetitive sequences within the mitogenomes of these fungi [76].

In this study, we compared the gene arrangements of four Lycoperdaceae species. Among the four mitogenome sequences, two species exhibited completely identical arrangements of PCGs, tRNA, and rRNA genes, which may represent the ancestral gene order inherited from the common ancestor of Lycoperdaceae species. Simultaneously, synteny analysis revealed that the two *Calvatia* species possessed an identical number of homologous regions in congruent arrangement. This finding corresponds to the phenomenon observed in the gene rearrangement analysis. In L. pratense, homologous region D (26,092–30060 bp) was located on the reverse strand of its mitogenome, containing *trnS1*, *trnE*, *trnM1*, *trnH*, *trnM2*, *trnK*, *trnR*, *trnL*, and *atp9* (Fig. 8). The presence of a cluster of nine consecutive genes located on the antisense strand in *L. pratense* has also been observed in *Leucoagaricus naucinus* (Agaricaceae). Furthermore, the last nine genes of a set of twelve consecutive genes in *L. naucinus* are identical to those found in *L. pratense*. More notably, similar phenomena were also observed in phylogenetically distant *Boletaceae* species. Although the number of contiguous gene clusters on the reverse strand varied among *Xerocomus impolitus*, *Tylopilus plumbeoviolaceoides*, *Aureoboletus raphanaceus*, and *Lanmaoa macrocarpa*, all species possessed a contiguous cluster composed of the same set of 16 genes: *rns*, *trnS*, *trnM*, *trnY*, *nad3*, *nad2*, *nad6*, *nad4*, *nad5*, *nad4L*, *cox3*, *cob*, *trnI*, *nad1*, *trnR*, and *trnC*.

In this study, we compared the gene arrangements of four Lycoperdaceae species. Among the four mitogenome sequences, two species exhibited completely identical arrangements of PCGs, tRNA, and rRNA genes, which may represent the ancestral gene order inherited from the common ancestor of Lycoperdaceae species. Simultaneously, synteny analysis revealed that the two *Calvatia* species possessed an identical number of homologous regions in congruent arrangement. This finding corresponds to the phenomenon observed in the gene rearrangement analysis. In *L*. *pratense*, homologous region D (26,092–30060 bp) was located on the reverse strand of its mitogenome, containing *trnS1*, *trnE*, *trnM1*, *trnH*, *trnM2*, *trnK*, *trnR*, *trnL*, and *atp9* (Fig. 8). The presence of a cluster of nine consecutive genes located on the antisense strand in *L. pratense* has also been observed in *Leucoagaricus naucinus* (Agaricaceae). Furthermore, the last nine genes of a set of twelve consecutive genes in *L. naucinus* are identical to those found in *L. pratense*. More notably, similar phenomena were also observed in phylogenetically distant Boletaceae species. Although the number of contiguous gene clusters on the reverse strand varied among *Xerocomus impolitus*, *Tylopilus plumbeoviolaceoides*, *Aureoboletus raphanaceus*, and *Lanmaoa macrocarpa*, all species possessed a contiguous cluster composed of the same set of 16 genes: *rns*, *trnS*, *trnM*, *trnY*, *nad3*, *nad2*, *nad6*, *nad4*, *nad5*, *nad4L*, *cox3*, *cob*, *trnI*, *nad1*, *trnR*, and *trnC*.

In terms of the evolutionary relationship between gasteroid fungi and Agaricales, the hypothesis of regressive evolution exists. It is believed that encapsulation of spores in the body by gasteromycetation protects the spores from death due to desiccation. Many fungi with very different appearances undergo gasteromycetization when faced with desiccation stress, since this process represents the optimal solution for spore protection under such conditions [77]. The phylogenetic tree reconstructed in this study includes several species of gasteroid fungi from the families Pisolithaceae, Rhizopogonaceae, and Lycoperdaceae. As the phylogenetic tree branches diverge, a decreasing trend is observed in the number of introns within the mitogenomes of Boletales species. The specific intron counts are as follows: Rhizopogonaceae with 16 and 20 introns, Pisolithaceae with 3 and 4 introns, Boletinellaceae with 4 introns, Paxillaceae with 4 introns, and Boletaceae with 0 and 2 introns. The above conclusion is consistent with the intron loss phenomenon observed in eukaryotes in prior studies [78–80]. Furthermore, the Boletaceae species within the reconstructed phylogeny exhibit structural characteristics typical of Agaricales, such as cap and stipe morphology, while simultaneously displaying distinctive features, notably a tubular hymenophore structure instead of the lamellate configuration characteristic of most Agaricales. Previous studies have confirmed that during the morphological evolution of Boletaceae species, multiple taxa have undergone gasteromycetation as an irreversible evolutionary trajectory [81–83]. Therefore, the Boletaceae species examined in this phylogenetic study exhibit evidence of intron loss during their evolution. Furthermore, their macro-morphological characteristics suggest the potential for future gasteromycetation in some lineages. Additionally, species within the same genus of gasteroid fungi in the order Boletales exhibit conserved intron numbers, a pattern also observed in the phylogenetically distant family Lycoperdaceae.

In summary, mitochondrial genes serve as reliable tools for studying the phylogenetic relationships within the Lycoperdaceae family. Fungi are widely distributed globally and have complex taxonomic systems. Advancing research necessitates the acquisition of more fungal mitogenomes to elucidate the origins, evolutionary pathways, and genetic diversity of fungi.

## Conclusions

In this study, the mitogenomes of four Lycoperdaceae species (*L*. *perlatum*, *L*. *pratense*, *C*. *caatinguensis*, and *C*. *boninensis*) were sequenced and annotated. These four mitogenomes ranged from 52,694 bp to 62,146 bp in size. The four newly sequenced mitogenomes had identical gene order: 42 genes, including 15 PCGs, 2 rRNAs, and 25 tRNAs. The mitochondrial genomic features of four Lycoperdaceae species, including gene content, genome size, gene arrangement, nucleotide composition, protein-coding gene (PCG) codon usage bias, and tRNA secondary structures, were comparatively analyzed. Pairwise comparisons of the four mitochondrial genomes revealed that introns contributed most significantly to length variation. Differences in both intron quantity and ORF content were identified as the primary drivers of mitochondrial genome size variation. The intron dynamics of *cox1* and *cob* genes were compared across eight agaricoid species of Agaricales and two gastroid species of Boletales (*Pisolithus* spp.), revealing frequent intron loss and gain events, as well as potential intron transfer phenomena during evolution. Boletales species exhibited a progressive decrease in intron numbers, with closely related species within the same genus maintaining similar intron quantities. In addition, we performed a phylogenetic analysis of Lycoperdaceae, which was used to determine the phylogenetic relationships between Lycoperdaceae spp. Based on the phylogenetic tree, *C*. *boninensis* and *C*. *caatinguensis* are sister species and closely related, and Lycoperdaceae and Agaricaceae are sister groups with high homology ratings. This study provides the first record of mitogenomes from Lycoperdaceae, thus providing a foundation for studying the evolution, genetics, and taxonomy of this important family and related fungal group.

## Electronic supplementary material

Below is the link to the electronic supplementary material.

Supplementary Material 1

Supplementary Material 2

## Data Availability

The complete mitogenomes of Lycoperdon perlatum, Lycoperdon pratense, Calvatia boninensis, and Calvatia caatinguensis were deposited in the GenBank database under the accession numbers PP690777, PP697973, PP670003, and PP840861, respectively.

## Abbreviations

mitogenome: Mitochondrial genome
nuc-ssu-rDNA: Nuclear small subunit ribosomal DNA
*ITS1*: Internal Transcribed spacers 1
LSU-rDNA: Large subunit ribosomal DNA
nrLSU: Nuclear ribosomal DNA large subunit
*rpb2*: RNA polymerase II second largest subunit
*tef1*-*α*: Eukaryotic translation elongation factor 1 alpha 1
*rpb1*: RNA polymerase II largest subunit
LSU: Large subunit
PCGs: Protein coding genes
tRNA: Transfer RNA
rRNA: Ribosomal RNA
AT: Adenine–thymine
GC: Guanine-cytosine
Ks: Synonymous substitution rate
Ka: Nonsynonymous substitution rate
K2P: Kimura-2-parameter
15PCG12: First and second codon positions of the 15 Protein Coding Genes
15PCG12R: First and second codon positions of the 15 Protein Coding Genes and 2 Ribosomal RNA genes.
AA: Amino acid sequences of the 15 Protein Coding Genes
ML: Maximum likelihood
BI: Bayesian inference
bp: Base pair
*atp6*: ATPase subunit 6
*atp8*: ATPase subunit 8
*atp9*: ATPase subunit 9
*cob*: Cytochrome b
*cox1*–*3*: Cytochrome oxidase subunit 1–3
*nad1*–*6*: NADH dehydrogenase subunit 1–6
*nad4L*: NADH dehydrogenase subunit 4 L
*rnl*: Large subunit ribosomal RNA
*rns*: Small subunit ribosomal RNA
Pcl: Position classes
