# Supplementary material for: Comparative analysis of mitochondrial genomes in lycoperdaceae fungi reveals intron dynamics and phylogenetic relationships
Source: BMC Genomics. 2025 Aug 11;26:742. doi: 10.1186/s12864-025-11911-4 (PMC12341119; doi:10.1186/s12864-025-11911-4)
Supplement: Supplementary file 1 — Supplementary Material 1 [file 12864_2025_11911_MOESM1_ESM.docx]

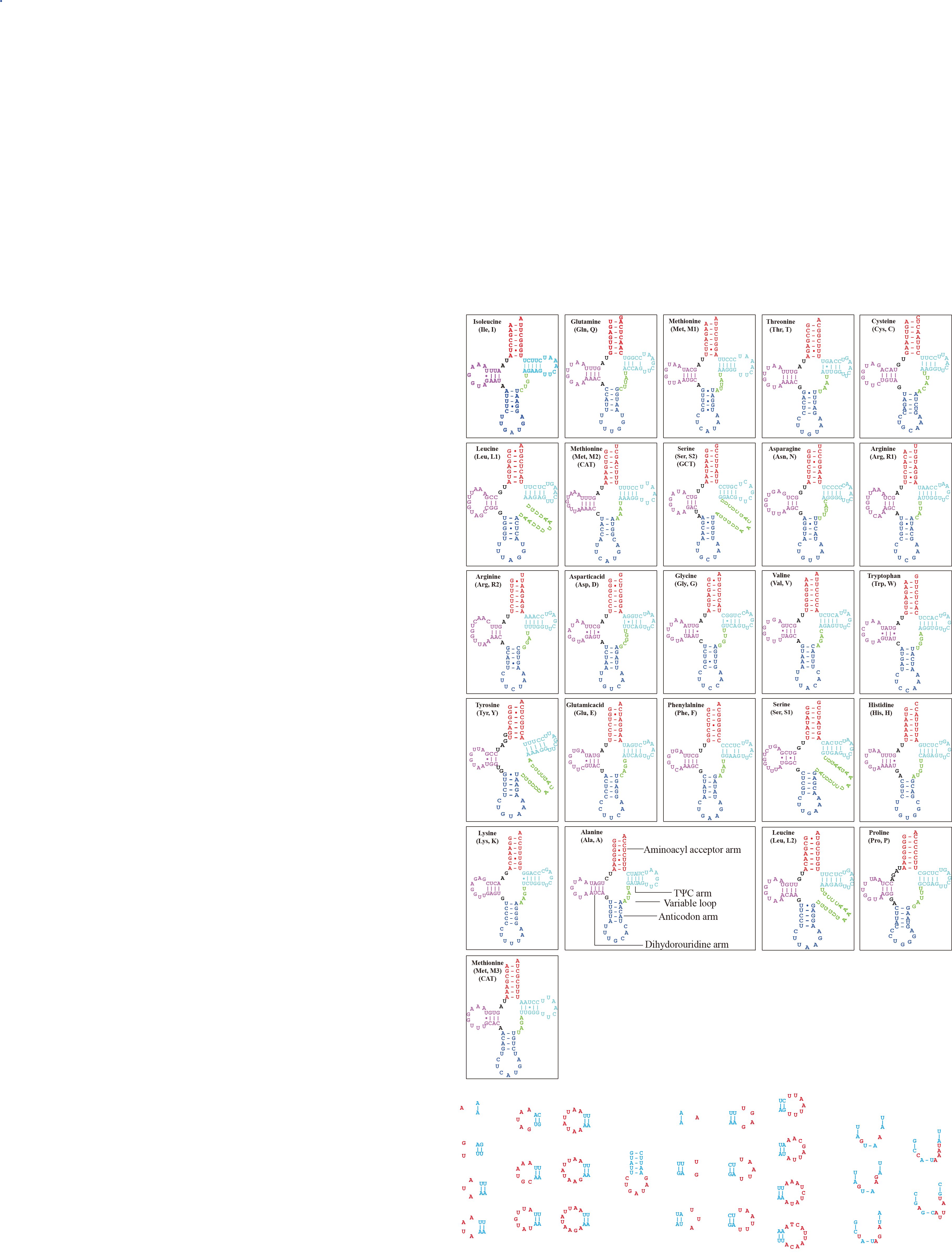
**Secondary structure diagram of tRNA in the other three species**

**Figure S1.** Predicted secondary structures of the 25 tRNAs of the *Calvatia caatinguensis* mitogenome.


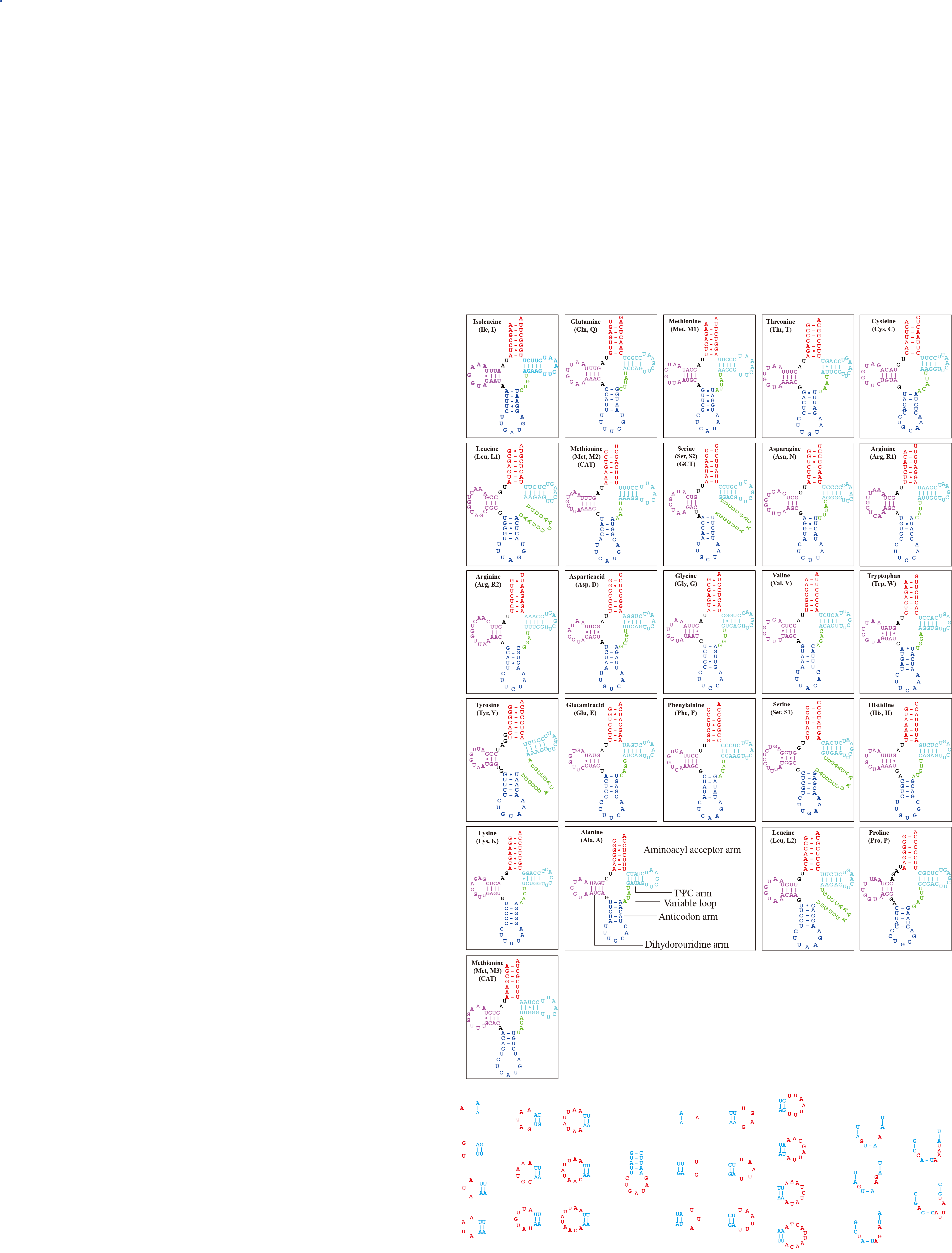


**Figure S2.** Predicted secondary structures of the 25 tRNAs of the *Calvatia boninensis* mitogenome.


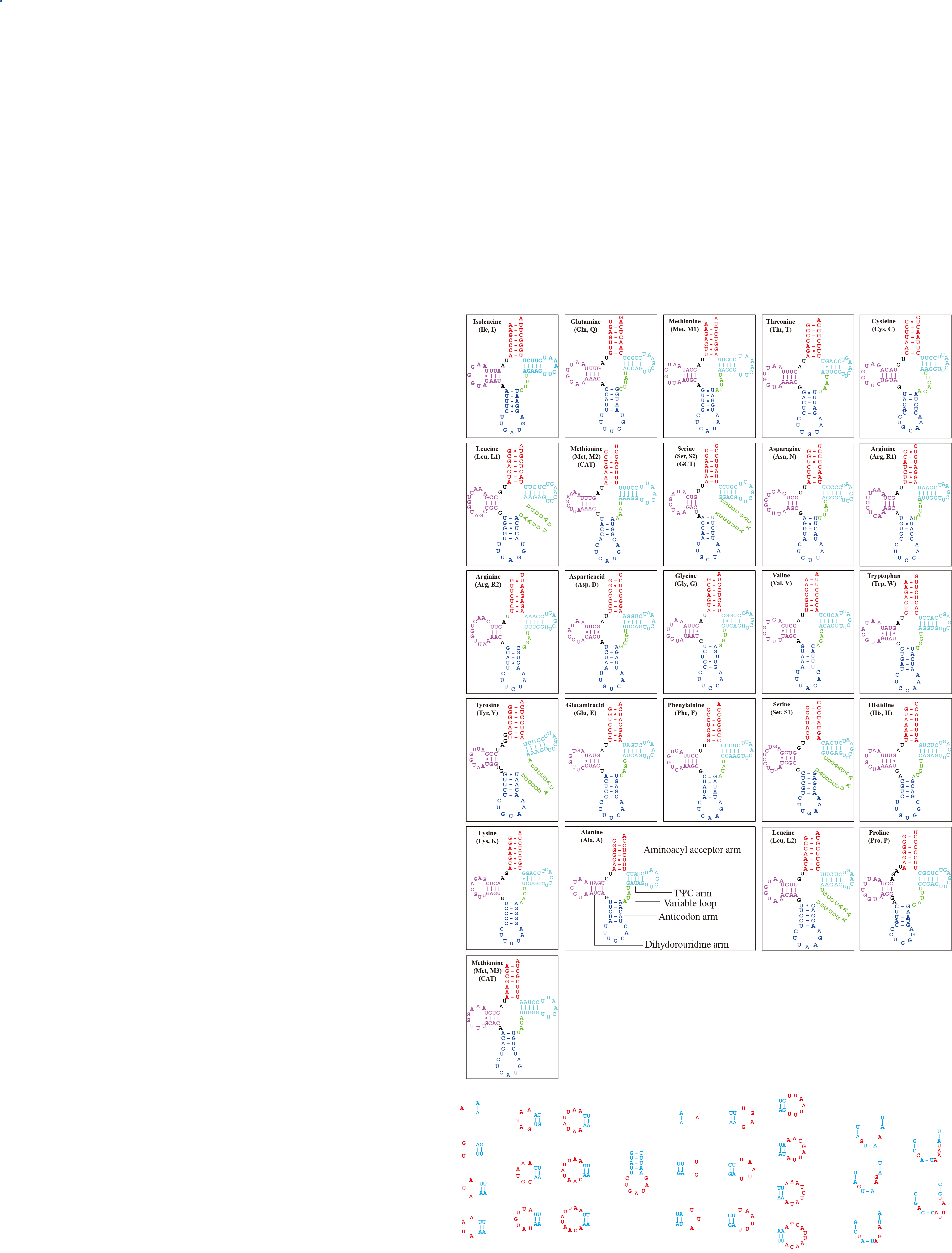


**Figure S3.** Predicted secondary structures of the 25 tRNAs of the *Lycoperdon pratense* mitogenome.

**
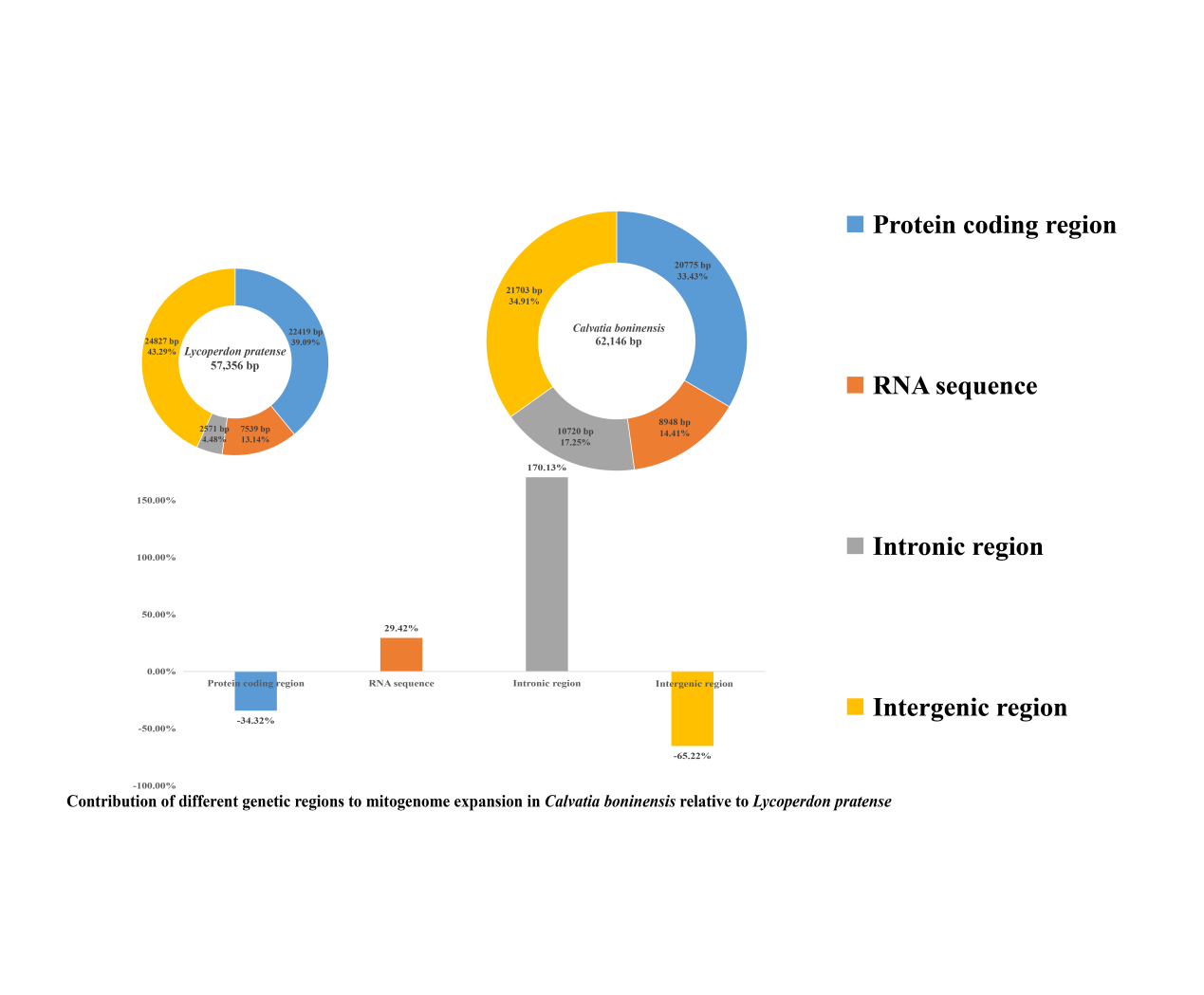
**

**Figure S4.** Contribution of different genetic regions to mitogenome expansion in *Calvatia boninensis* relative to *Lycoperdon pratense*


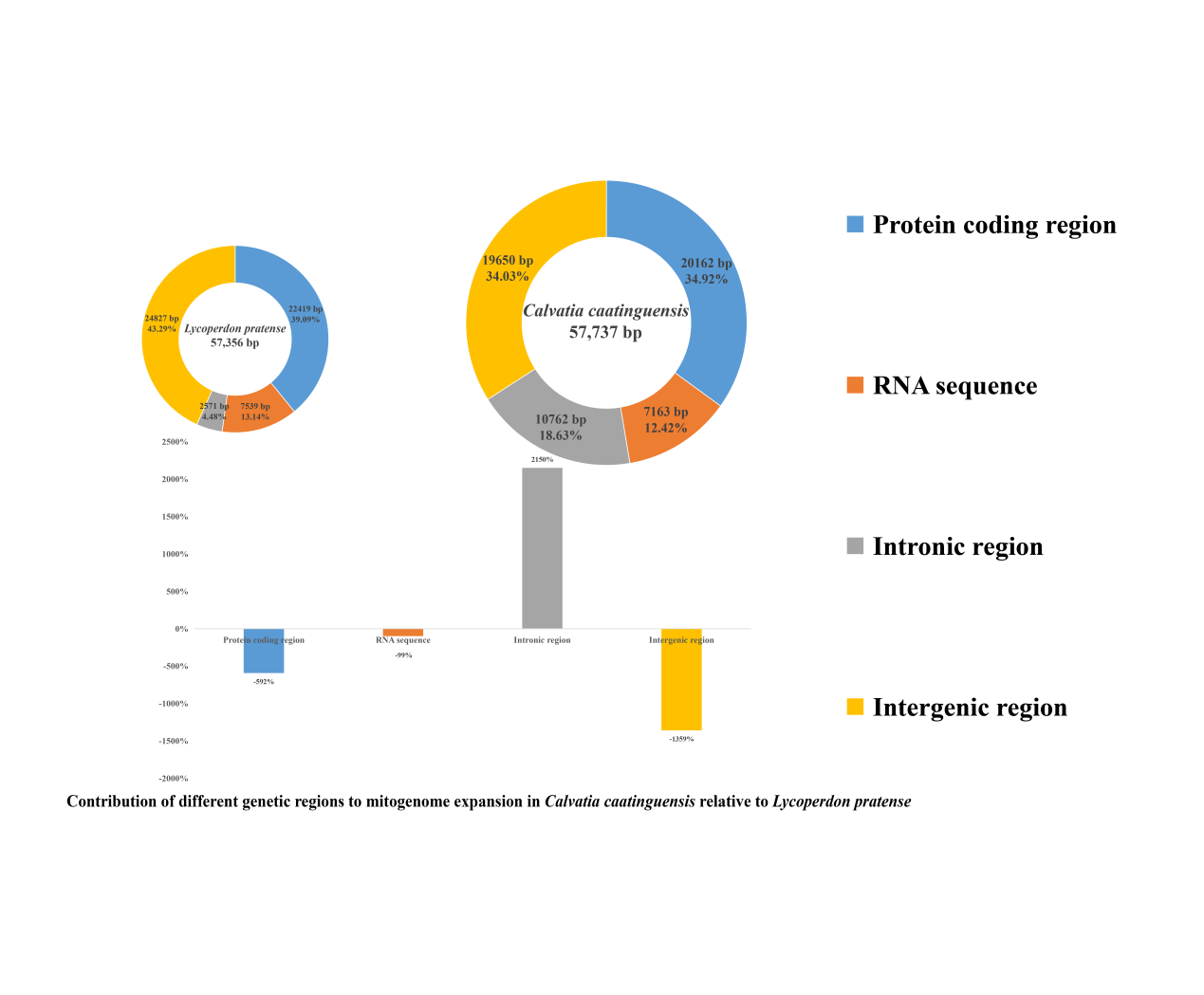


**Figure S5.** Contribution of different genetic regions to mitogenome expansion in *Calvatia caatinguensis* relative to *Lycoperdon pratense*

**
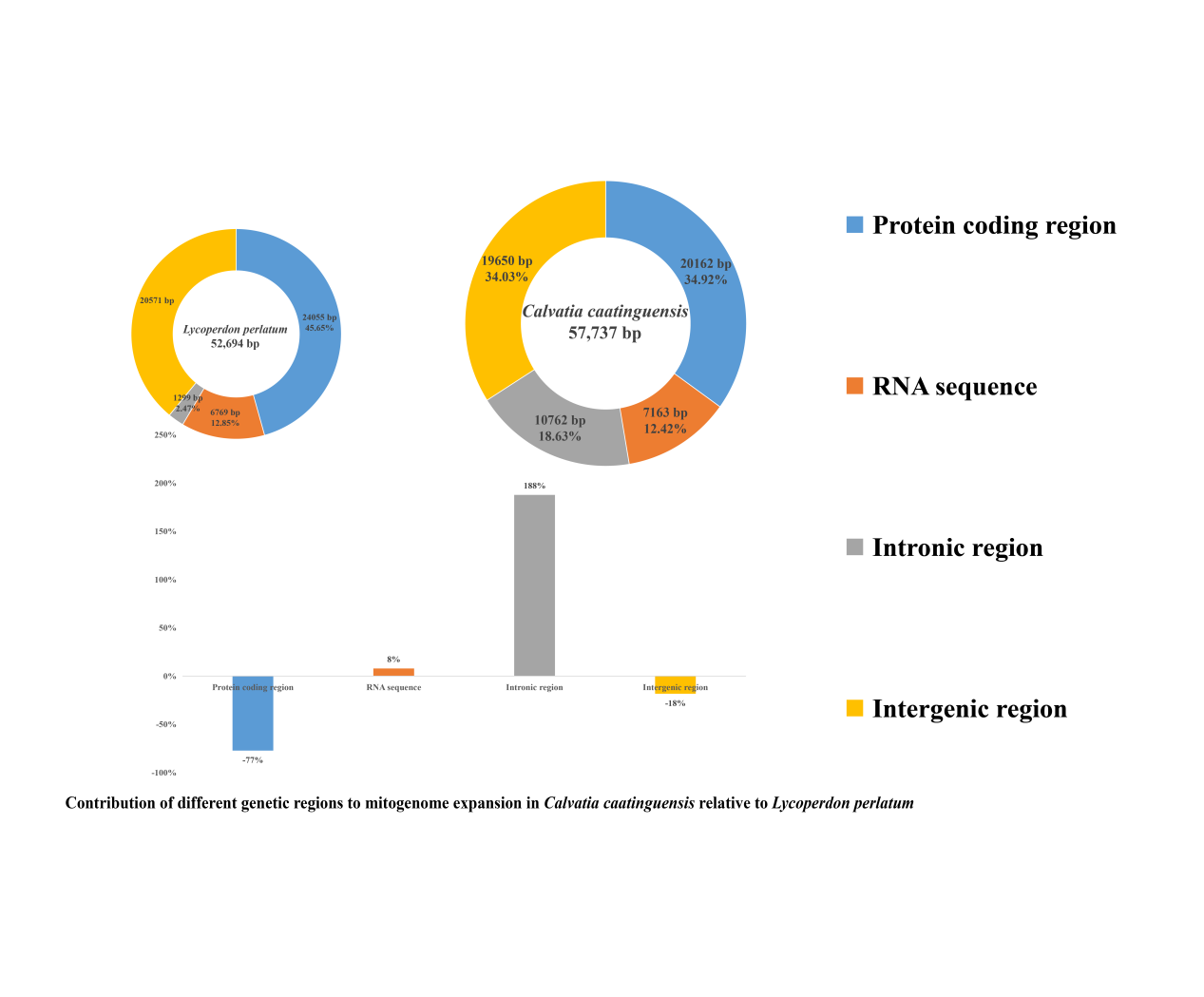
Figure S6.** Contribution of different genetic regions to mitogenome expansion in *Calvatia boninensis* relative to *Lyceperdon perlatum*

**Phylogenetic tree constructed from other datasets**


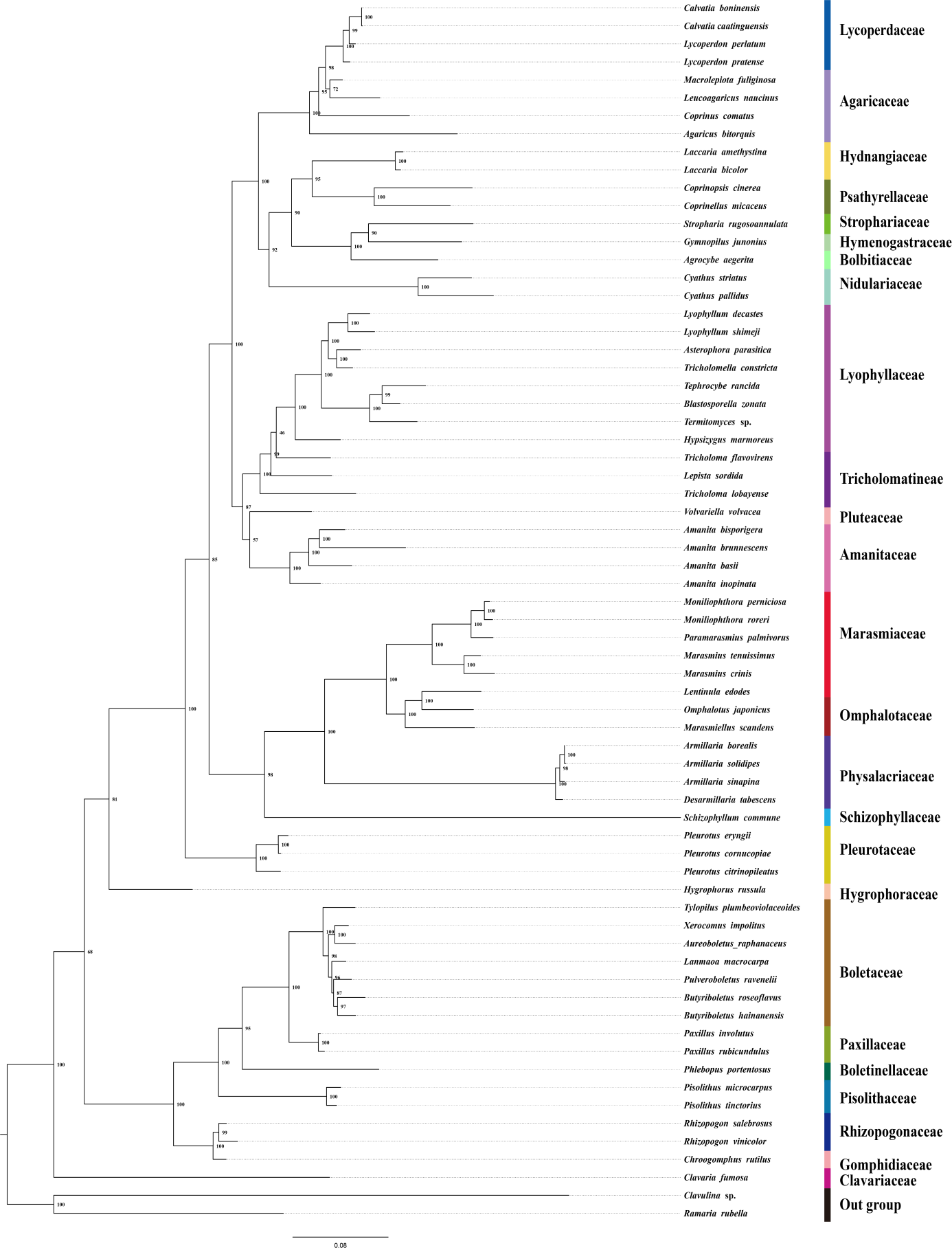
**Figure S7.** The phylogenetic tree of AA dataset constructed using iqtree. The best-fit evolutionary model for the mitochondrial gene dataset was determined to be LG+I+G+F using the PartitionFinder tool in PhyloSuite v1.2.2.


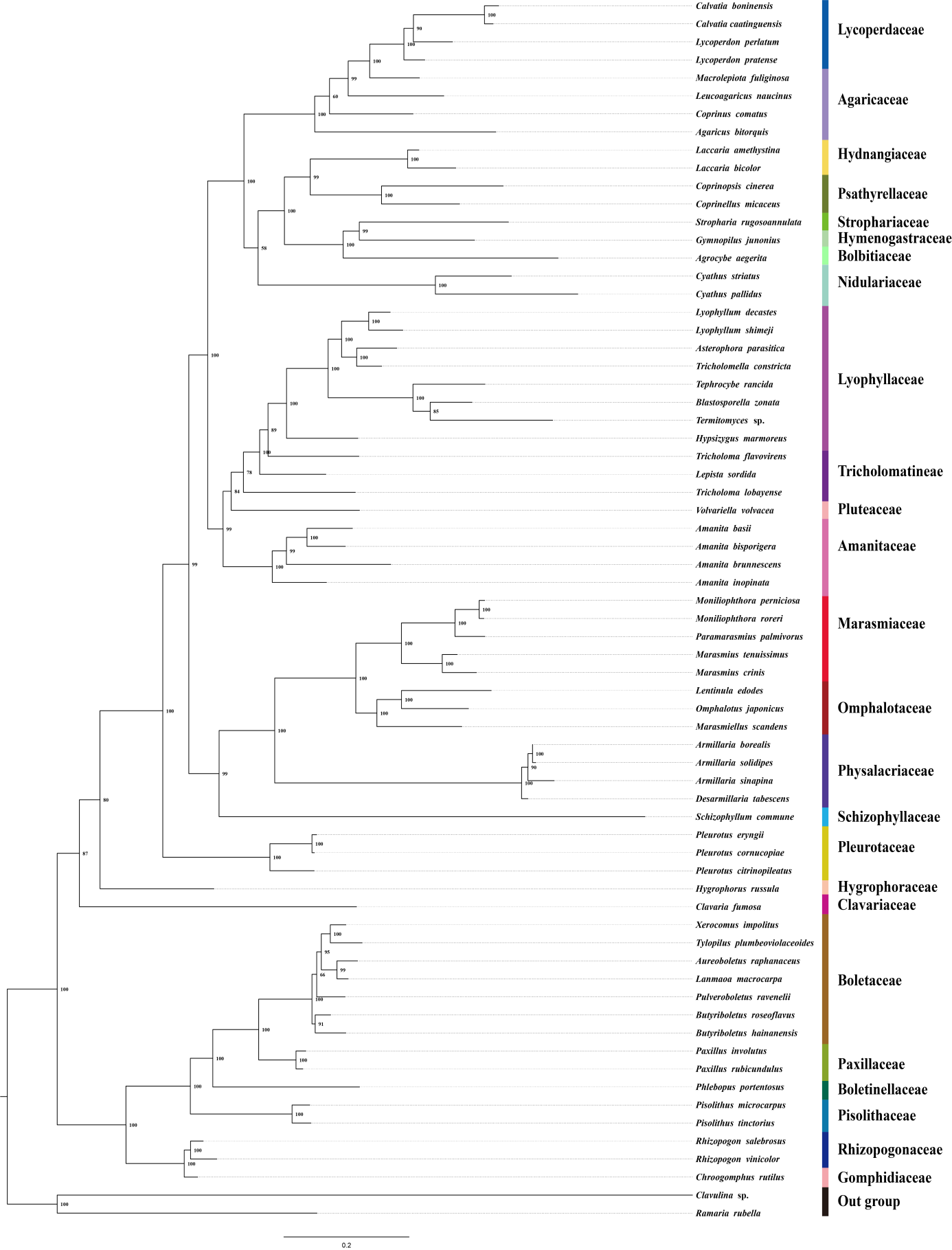
**Figure S8.** The Phylogenetic tree of 15PCG12R dataset constructed using iqtree. The best-fit evolutionary model for the mitochondrial gene dataset was determined to be GTR+I+G using the PartitionFinder tool in PhyloSuite v1.2.2.


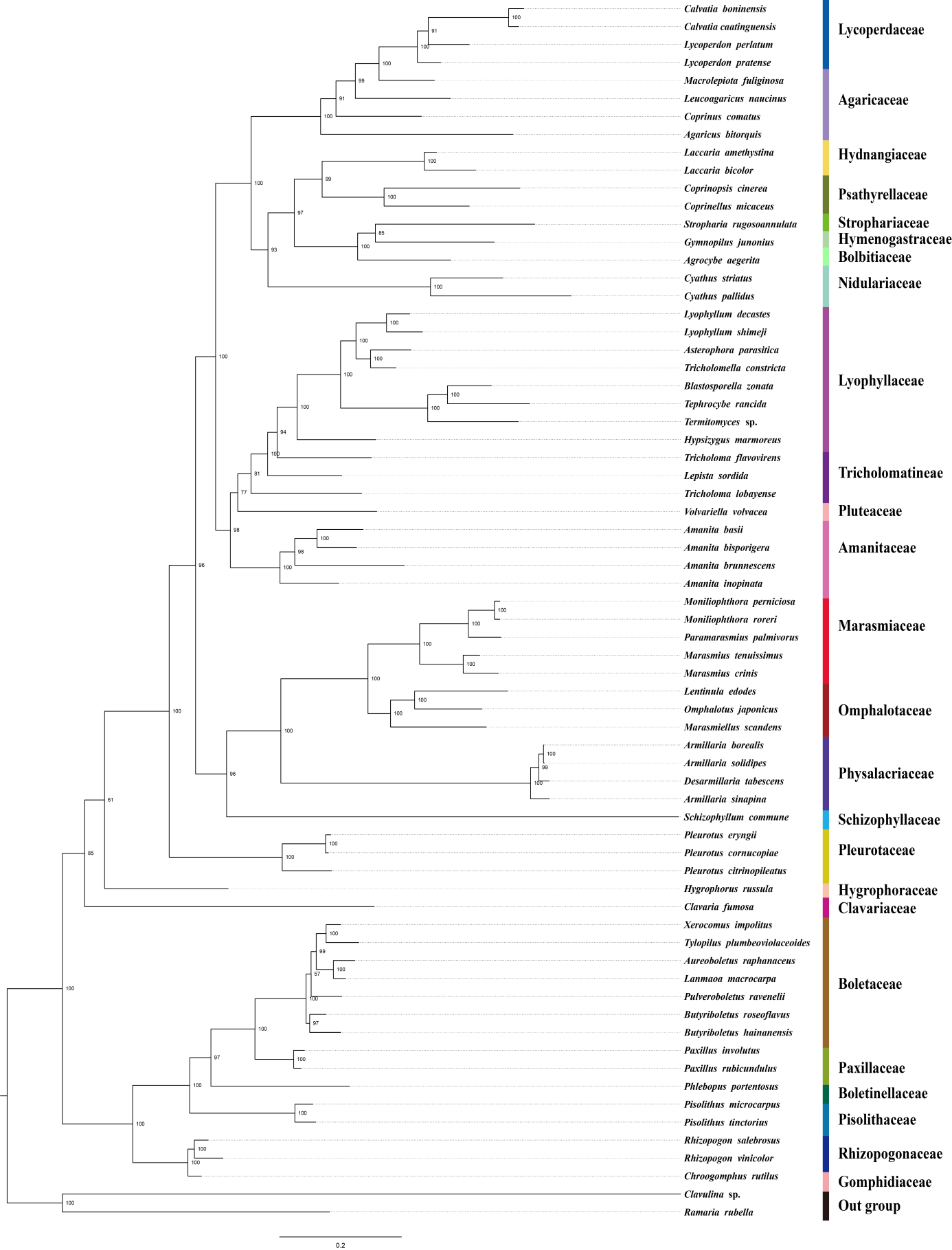
**Figure S9.** The Phylogenetic tree of 15PCG12 dataset constructed using iqtree. The best-fit evolutionary model for the mitochondrial gene dataset was determined to be GTR+I+G using the PartitionFinder tool in PhyloSuite v1.2.2.


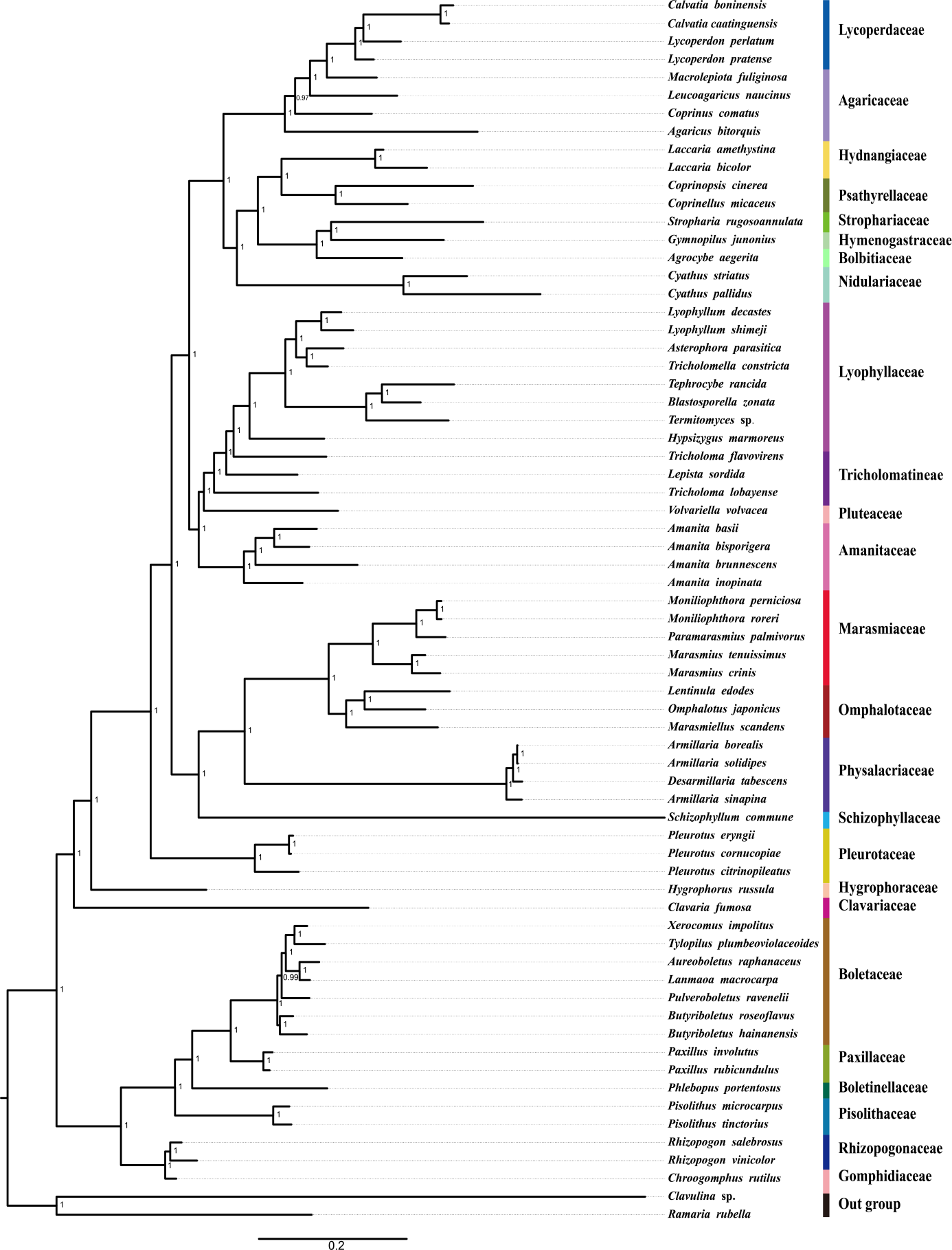
**Figure S10.** The Phylogenetic tree of 15PCG12 dataset constructed using MrBayes. The best-fit evolutionary model for the mitochondrial gene dataset was determined to be GTR+I+G using the PartitionFinder tool in PhyloSuite v1.2.2.

**
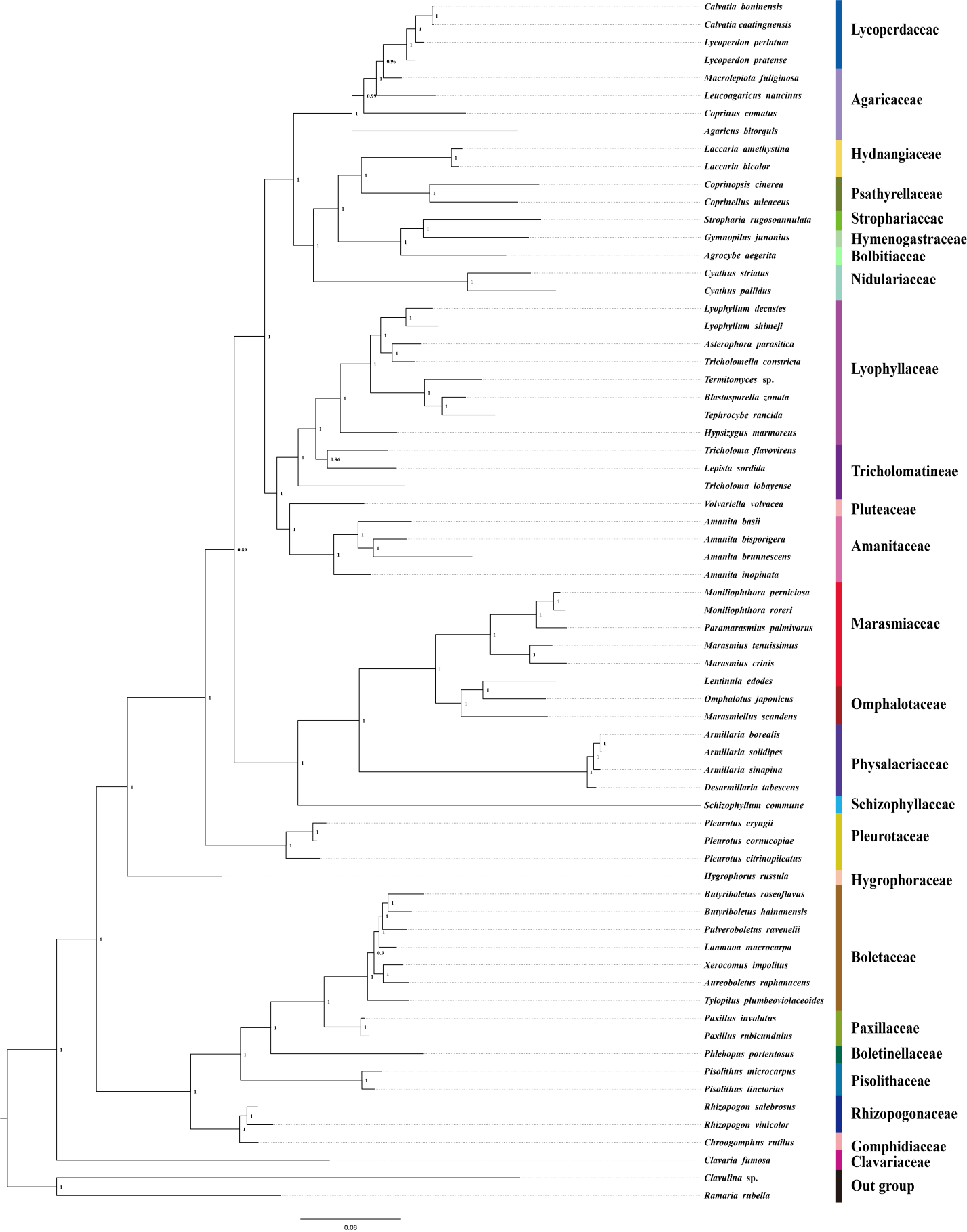
Figure S11.** The Phylogenetic tree of AA dataset constructed using MrBayes. The best-fit evolutionary model for the mitochondrial gene dataset was determined to be LG+I+G+F using the PartitionFinder tool in PhyloSuite v1.2.2.
