# Supplementary material for: Comparative analysis of mitochondrial genomes in lycoperdaceae fungi reveals intron dynamics and phylogenetic relationships
Source: BMC Genomics. 2025 Aug 11;26:742. doi: 10.1186/s12864-025-11911-4 (PMC12341119; doi:10.1186/s12864-025-11911-4)
Supplement: Supplementary file 2 — Supplementary Material 2 [file 12864_2025_11911_MOESM2_ESM.docx]

| **Table S1 Collecting information** | | | | |
| --- | --- | --- | --- | --- |
| Specie | Collector | Locus | Habitat | Time |
| *C. boninensis* | Xianyi Wang, Zhongyao Guo | Huaxi District, Guiyang City, Guizhou Province, China Altitude: 1177 meters Longitude: 106 ° 63 ′ 67 ″ Latitude: 26 ° 37 ′ 32 ″ | Found on decaying leaves | Jun, 2023 |
| *C. caatinguensis* | Jiawei Tao, Guangyin Xu, Guoyu Wang | Doupeng Mountain, Duyun City, Qiannan Prefecture, Guizhou Province, China Altitude: 1961 meters Longitude: 107 ° 31 ′ 19 ″ Latitude: 25 ° 51 ′ 26 ″ | Found on decaying leaves | Jun, 2023 |
| *L. perlatum* | Zhongyao Guo, Guangyin Xu, Jiawei Tao | Guizhou Province, China, Guiyang City, Guiyang Forest Park Altitude: 1169 meters Longitude: 106 ° 75 ′ 89 ″ Latitude: 25 ° 55 ′ 61 ″ | Found on decaying leaves | Jun, 2023 |
| *L. pratense* | Xianyi Wang, Zhongyao Guo, Guoyu Wang | Huaxi District, Guiyang City, Guizhou Province, China Altitude: 1177 meters Longitude: 106 ° 63 ′ 67 ″ Latitude: 26 ° 37 ′ 32 ″ | Found on lawn land | Jun, 2023 |

| **Table S2 The species information used for intron dynamics analysis in this study** | | | | | |
| --- | --- | --- | --- | --- | --- |
| **ID** | **Species** | **GenBank accession number** | **Family** | **genus** | **Source** |
| M1126 | *Lycoperdon perlatum* | PP690777 | Lycoperdaceae | *Lycoperdon* | This study |
| M817 | *Lycoperdon pratense* | PP697973 |  |  | This study |
| M842 | *Calvatia boninensis* | PP670003 |  | *Calvatia* | This study |
| M867 | *Calvatia caatinguensis* | PP840861 |  |  | This study |
| AB | *Agaricus bitorquis* | NC077556 | Agaricaceae | *Agaricus* | Ferandon et al. |
| CC | *Coprinus comatus* | NC059951 |  | *Coprinus* | Unpublished |
| MF | *Macrolepiota fuliginosa* | NC045202 |  | *Macrolepiota* | Unpublished |
| LN | *Leucoagaricus naucinus* | [MZ352929](https://doi.org/10.1080/23802359.2021.1970643) |  | *Leucoagaricus* | Li et al. |
| PM | *Pisolithus microcarpus* | NC054201 | Pisolithaceae | *Pisolithus* | Wu et al. |
| PT | *Pisolithus tinctorius* | NC054202 |  |  | Wu et al. |

| **Table S3 Start and stop codons analysis of 4 species from Lycoperdaceae** | | | | | | | | | | | | | | |  |  |
| --- | --- | --- | --- | --- | --- | --- | --- | --- | --- | --- | --- | --- | --- | --- | --- | --- |
| **Species** | ***cox1*** | | ***cox2*** | | ***cox3*** | | ***atp6*** | | ***atp8*** | | ***atp9*** | | ***nad1*** | |  |  |
|  | start | stop | start | stop | start | stop | start | stop | start | stop | start | stop | start | stop |  |  |
| ***Calvatia boninensis*** | ATG | TAA | ATG | TAA | ATG | TAA | ATT | TAA | ATG | TAA | TTA | TAA | TTA | TAA |  |  |
| ***Calvatia caatinguensis*** | ATG | TAG | ATG | TAA | ATG | TAA | ATG | TAA | ATA | TAA | TTA | TAA | ATA | TAA |  |  |
| ***Lycoperdon perlatum*** | ATG | TAA | ATG | TAG | ATG | TAA | ATA | TAA | ATT | TAA | ATA | TAA | ATA | TAA |  |  |
| ***Lycoperdon pratense*** | ATG | TAA | ATA | TAG | ATG | TAA | ATA | TAA | ATG | TAA | TAT | TAG | TTA | TAG |  |  |
|  | ***nad2*** | | ***nad3*** | | ***nad4*** | | ***nad4L*** | | ***nad5*** | | ***nad6*** | | ***cob*** | | ***rps3*** | |
|  | start | stop | start | stop | start | stop | start | stop | start | stop | start | stop | start | stop | start | stop |
|  | ATG | TAA | ATG | TAA | ATG | TAA | ATG | TAA | ATG | TAA | ATG | TAA | ATG | TAA | ATG | TAG |
|  | ATA | TAA | ATG | TAA | TTA | TAA | ATG | TAA | ATG | TAA | ATA | TAG | ATG | TAA | ATG | TAG |
|  | ATG | TAA | TTG | TAA | TTG | TAA | ATG | TAA | ATG | TAA | ATG | TAG | TTA | TAA | ATG | TAA |
|  | ATG | TAA | ATG | TAA | ATG | TAA | ATG | TAA | ATG | TAA | ATG | TAG | ATG | TAA | ATG | TAG |

| **Table S4 Local BLAST analysis of the Lycoperdaceae mitogenomes against themselves** | | | | | | | | | |
| --- | --- | --- | --- | --- | --- | --- | --- | --- | --- |
| Species | Identitie (%) | Aligned length (bp) | Mismatched bases (bp) | Gaps (bp) | from | to | from | to | evalue |
| *Calvatia boninensis* | 99.291% | 141 | 1 | 0 | 57808 | 57948 | 58928 | 59068 | 3.00E-68 |
| *Calvatia boninensis* | 99.291% | 141 | 1 | 0 | 58928 | 59068 | 57808 | 57948 | 3.00E-68 |
| *Calvatia boninensis* | 95.833% | 72 | 3 | 0 | 24673 | 24744 | 32295 | 32366 | 2.00E-26 |
| *Calvatia boninensis* | 95.833% | 72 | 3 | 0 | 32295 | 32366 | 24673 | 24744 | 2.00E-26 |
| *Calvatia boninensis* | 100% | 77 | 0 | 0 | 5154 | 5230 | 59232 | 59308 | 3.00E-34 |
| *Calvatia boninensis* | 100% | 77 | 0 | 0 | 59232 | 59308 | 5154 | 5230 | 3.00E-34 |
| *Calvatia boninensis* | 95.833% | 72 | 3 | 0 | 24673 | 24744 | 32295 | 32366 | 2.00E-26 |
| *Calvatia boninensis* | 95.833% | 72 | 3 | 0 | 32295 | 32366 | 24673 | 24744 | 2.00E-26 |
| *Calvatia boninensis* | 98.462% | 65 | 0 | 1 | 26488 | 26551 | 32311 | 32375 | 2.00E-25 |
| *Calvatia boninensis* | 98.462% | 65 | 0 | 1 | 32311 | 32375 | 26488 | 26551 | 2.00E-25 |
| *Calvatia boninensis* | 92.647% | 68 | 2 | 3 | 24677 | 24744 | 26478 | 26542 | 8.00E-20 |
| *Calvatia boninensis* | 92.647% | 68 | 2 | 3 | 26478 | 26542 | 24677 | 24744 | 8.00E-20 |
| *Calvatia boninensis* | 80.916% | 131 | 11 | 14 | 57600 | 57727 | 58805 | 58924 | 1.00E-18 |
| *Calvatia boninensis* | 80.916% | 131 | 11 | 14 | 58805 | 58924 | 57600 | 57727 | 1.00E-18 |
| *Calvatia boninensis* | 100% | 48 | 0 | 0 | 27125 | 27172 | 44432 | 44432 | 4.00E-18 |
| *Calvatia boninensis* | 100% | 48 | 0 | 0 | 44432 | 44432 | 27125 | 27172 | 4.00E-18 |
| *Calvatia boninensis* | 98.039% | 51 | 1 | 0 | 37452 | 37502 | 58700 | 58750 | 4.00E-18 |
| *Calvatia boninensis* | 98.039% | 51 | 1 | 0 | 58700 | 58750 | 37452 | 37502 | 4.00E-18 |
| *Calvatia boninensis* | 93.220% | 59 | 3 | 1 | 26245 | 26302 | 32156 | 32214 | 5.00E-17 |
| *Calvatia boninensis* | 93.220% | 59 | 3 | 1 | 32156 | 32214 | 26245 | 26302 | 5.00E-17 |
| *Calvatia boninensis* | 100% | 44 | 0 | 0 | 159 | 202 | 1532 | 1575 | 6.00E-16 |
| *Calvatia boninensis* | 100% | 44 | 0 | 0 | 1532 | 1575 | 159 | 202 | 6.00E-16 |
| *Calvatia boninensis* | 92.727% | 55 | 4 | 0 | 56689 | 56743 | 57439 | 57385 | 2.00E-15 |
| *Calvatia boninensis* | 92.727% | 55 | 4 | 0 | 57385 | 57439 | 56743 | 56689 | 2.00E-15 |
| *Calvatia boninensis* | 100% | 39 | 0 | 0 | 22691 | 22729 | 28144 | 28106 | 4.00E-13 |
| *Calvatia boninensis* | 100% | 39 | 0 | 0 | 28106 | 28144 | 22729 | 22691 | 4.00E-13 |
| *Calvatia boninensis* | 100% | 38 | 0 | 0 | 61464 | 61501 | 61501 | 61464 | 1.00E-12 |
| *Calvatia boninensis* | 100% | 36 | 0 | 0 | 57582 | 57617 | 61003 | 60968 | 2.00E-11 |
| *Calvatia boninensis* | 100% | 36 | 0 | 0 | 60968 | 61003 | 57617 | 57582 | 2.00E-11 |
| *Calvatia caatinguensis* | 99.291% | 141 | 1 | 0 | 53378 | 53518 | 54498 | 54638 | 3.00E-68 |
| *Calvatia caatinguensis* | 99.291% | 141 | 1 | 0 | 54498 | 54638 | 53378 | 53518 | 3.00E-68 |
| *Calvatia caatinguensis* | 92% | 100 | 4 | 4 | 23865 | 23963 | 25397 | 25493 | 1.00E-32 |
| *Calvatia caatinguensis* | 92% | 100 | 4 | 4 | 25397 | 25493 | 23865 | 23963 | 1.00E-32 |
| *Calvatia caatinguensis* | 100% | 56 | 0 | 0 | 18381 | 18436 | 34811 | 34866 | 1.00E-22 |
| *Calvatia caatinguensis* | 100% | 56 | 0 | 0 | 34811 | 34866 | 18381 | 18436 | 1.00E-22 |
| *Calvatia caatinguensis* | 80.916% | 131 | 11 | 14 | 53170 | 53297 | 54375 | 54494 | 9.00E-19 |
| *Calvatia caatinguensis* | 80.916% | 131 | 11 | 14 | 54375 | 54494 | 53170 | 53297 | 9.00E-19 |
| *Calvatia caatinguensis* | 100% | 48 | 0 | 0 | 26061 | 26108 | 41481 | 41528 | 3.00E-18 |
| *Calvatia caatinguensis* | 100% | 48 | 0 | 0 | 41481 | 41528 | 26061 | 26108 | 3.00E-18 |
| *Calvatia caatinguensis* | 92.727% | 55 | 4 | 0 | 52267 | 52321 | 53017 | 52963 | 2.00E-15 |
| *Calvatia caatinguensis* | 92.727% | 55 | 4 | 0 | 52963 | 53017 | 52321 | 52267 | 2.00E-15 |
| *Calvatia caatinguensis* | 100% | 38 | 0 | 0 | 57028 | 57065 | 57065 | 57028 | 1.00E-12 |
| *Calvatia caatinguensis* | 100% | 37 | 0 | 0 | 53151 | 53187 | 56568 | 56532 | 4.00E-12 |
| *Calvatia caatinguensis* | 100% | 37 | 0 | 0 | 56532 | 56568 | 53187 | 53151 | 4.00E-12 |
| *Lycoperdon perlatum* | 96.324% | 136 | 4 | 1 | 35775 | 35909 | 9542 | 9677 | 3.00E-58 |
| *Lycoperdon perlatum* | 94.167% | 120 | 3 | 4 | 8518 | 8633 | 13359 | 13478 | 2.00E-45 |
| *Lycoperdon perlatum* | 94.167% | 120 | 3 | 4 | 13359 | 13478 | 8518 | 8633 | 2.00E-45 |
| *Lycoperdon perlatum* | 94.118% | 102 | 5 | 1 | 13388 | 13488 | 27215 | 27316 | 9.00E-38 |
| *Lycoperdon perlatum* | 94.118% | 102 | 5 | 1 | 27215 | 27316 | 13388 | 13488 | 9.00E-38 |
| *Lycoperdon perlatum* | 94.937% | 79 | 2 | 2 | 12864 | 12941 | 44180 | 44103 | 3.00E-28 |
| *Lycoperdon perlatum* | 94.937% | 79 | 2 | 2 | 44103 | 44180 | 12941 | 12864 | 3.00E-28 |
| *Lycoperdon perlatum* | 89.130% | 92 | 5 | 5 | 8547 | 8633 | 27215 | 27306 | 2.00E-24 |
| *Lycoperdon perlatum* | 89.130% | 92 | 5 | 5 | 27215 | 27306 | 8547 | 8633 | 2.00E-24 |
| *Lycoperdon perlatum* | 93.220% | 59 | 4 | 0 | 9755 | 9813 | 13491 | 13549 | 1.00E-17 |
| *Lycoperdon perlatum* | 93.220% | 59 | 4 | 0 | 13491 | 13549 | 9755 | 9813 | 1.00E-17 |
| *Lycoperdon perlatum* | 95.918% | 49 | 2 | 0 | 12669 | 12717 | 23911 | 23863 | 2.00E-15 |
| *Lycoperdon perlatum* | 97.826% | 46 | 0 | 1 | 23863 | 23908 | 12717 | 12672 | 2.00E-15 |
| *Lycoperdon perlatum* | 84.810% | 79 | 7 | 5 | 24203 | 24276 | 46879 | 46957 | 7.00E-14 |
| *Lycoperdon perlatum* | 93.617% | 47 | 3 | 0 | 12876 | 12922 | 44956 | 44910 | 1.00E-12 |
| *Lycoperdon perlatum* | 95.455% | 44 | 2 | 0 | 44122 | 44165 | 44910 | 44953 | 1.00E-12 |
| *Lycoperdon perlatum* | 95.455% | 44 | 2 | 0 | 44910 | 44953 | 12922 | 12879 | 1.00E-12 |
| *Lycoperdon perlatum* | 95.455% | 44 | 2 | 0 | 44910 | 44953 | 44122 | 44165 | 1.00E-12 |
| *Lycoperdon perlatum* | 100% | 36 | 0 | 0 | 33304 | 33339 | 33339 | 33304 | 1.00E-11 |
| *Lycoperdon perlatum* | 100% | 35 | 0 | 0 | 22143 | 22177 | 26925 | 26959 | 4.00E-11 |
| *Lycoperdon perlatum* | 100% | 35 | 0 | 0 | 26925 | 26959 | 22143 | 22177 | 4.00E-11 |
| *Lycoperdon pratense* | 87.151% | 179 | 17 | 6 | 29811 | 29986 | 30921 | 31096 | 5.00E-51 |
| *Lycoperdon pratense* | 87.151% | 179 | 17 | 6 | 30921 | 31096 | 29811 | 29986 | 5.00E-51 |
| *Lycoperdon pratense* | 98.462% | 65 | 1 | 0 | 5826 | 5890 | 52599 | 52535 | 5.00E-26 |
| *Lycoperdon pratense* | 100% | 62 | 0 | 0 | 14997 | 15058 | 16013 | 15952 | 5.00E-26 |
| *Lycoperdon pratense* | 100% | 62 | 0 | 0 | 15952 | 16013 | 15058 | 14997 | 5.00E-26 |
| *Lycoperdon pratense* | 98.462% | 65 | 1 | 0 | 52535 | 52599 | 5890 | 5826 | 5.00E-26 |
| *Lycoperdon pratense* | 97.015% | 67 | 2 | 0 | 48051 | 48117 | 50936 | 50870 | 2.00E-25 |
| *Lycoperdon pratense* | 97.015% | 67 | 2 | 0 | 50870 | 50936 | 48117 | 48051 | 2.00E-25 |
| *Lycoperdon pratense* | 98.438% | 64 | 0 | 1 | 2719 | 2782 | 50870 | 50932 | 7.00E-25 |
| *Lycoperdon pratense* | 98.438% | 64 | 0 | 1 | 50870 | 50932 | 2719 | 2782 | 7.00E-25 |
| *Lycoperdon pratense* | 94.366% | 71 | 3 | 1 | 2712 | 2782 | 48124 | 48055 | 9.00E-24 |
| *Lycoperdon pratense* | 94.366% | 71 | 3 | 1 | 48055 | 48124 | 2782 | 2712 | 9.00E-24 |
| *Lycoperdon pratense* | 95.588% | 68 | 1 | 2 | 15952 | 16019 | 16101 | 16166 | 9.00E-24 |
| *Lycoperdon pratense* | 95.588% | 68 | 1 | 2 | 16101 | 16166 | 15952 | 16019 | 9.00E-24 |
| *Lycoperdon pratense* | 89.873% | 79 | 5 | 3 | 40408 | 40485 | 29519 | 29443 | 5.00E-21 |
| *Lycoperdon pratense* | 95.161% | 62 | 1 | 2 | 14997 | 15058 | 16160 | 16101 | 2.00E-20 |
| *Lycoperdon pratense* | 95.161% | 62 | 1 | 2 | 16101 | 16160 | 15058 | 14997 | 2.00E-20 |
| *Lycoperdon pratense* | 90.141% | 71 | 6 | 1 | 8987 | 9057 | 12315 | 12384 | 9.00E-19 |
| *Lycoperdon pratense* | 90.141% | 71 | 6 | 1 | 12315 | 12384 | 8987 | 9057 | 9.00E-19 |
| *Lycoperdon pratense* | 88% | 75 | 8 | 1 | 12387 | 12461 | 12555 | 12628 | 1.00E-17 |
| *Lycoperdon pratense* | 88% | 75 | 8 | 1 | 12555 | 12628 | 12387 | 12461 | 1.00E-17 |
| *Lycoperdon pratense* | 88.889% | 72 | 2 | 6 | 2416 | 2484 | 9057 | 8989 | 1.00E-16 |
| *Lycoperdon pratense* | 88.889% | 72 | 2 | 6 | 8989 | 9057 | 2484 | 2416 | 1.00E-16 |
| *Lycoperdon pratense* | 100% | 44 | 0 | 0 | 13127 | 13170 | 16153 | 16110 | 5.00E-16 |
| *Lycoperdon pratense* | 100% | 44 | 0 | 0 | 16110 | 16153 | 13170 | 13127 | 5.00E-16 |
| *Lycoperdon pratense* | 97.826% | 46 | 0 | 1 | 13127 | 13171 | 15004 | 15049 | 7.00E-15 |
| *Lycoperdon pratense* | 97.826% | 46 | 0 | 1 | 15004 | 15049 | 13127 | 13171 | 7.00E-15 |
| *Lycoperdon pratense* | 97.826% | 46 | 0 | 1 | 13127 | 13171 | 16006 | 15961 | 7.00E-15 |
| *Lycoperdon pratense* | 97.826% | 46 | 0 | 1 | 15961 | 16006 | 13171 | 13127 | 7.00E-15 |
| *Lycoperdon pratense* | 92.593% | 54 | 3 | 1 | 24182 | 24234 | 24759 | 24706 | 2.00E-14 |
| *Lycoperdon pratense* | 92.727% | 55 | 1 | 3 | 24706 | 24759 | 24234 | 24182 | 2.00E-14 |
| *Lycoperdon pratense* | 100% | 39 | 0 | 0 | 17333 | 17371 | 18571 | 18609 | 3.00E-13 |
| *Lycoperdon pratense* | 100% | 39 | 0 | 0 | 18571 | 18609 | 17333 | 17371 | 3.00E-13 |
| *Lycoperdon pratense* | 100% | 39 | 0 | 0 | 48984 | 49022 | 21689 | 21727 | 3.00E-13 |
| *Lycoperdon pratense* | 97.561% | 41 | 1 | 0 | 17176 | 17216 | 18774 | 18814 | 1.00E-12 |
| *Lycoperdon pratense* | 97.561% | 41 | 1 | 0 | 18774 | 18814 | 17176 | 17216 | 1.00E-12 |
| *Lycoperdon pratense* | 95.349% | 43 | 2 | 0 | 13526 | 13568 | 18716 | 18674 | 4.00E-12 |
| *Lycoperdon pratense* | 95.349% | 43 | 2 | 0 | 18674 | 18716 | 13568 | 13526 | 4.00E-12 |
| *Lycoperdon pratense* | 97.5% | 40 | 1 | 0 | 17494 | 17533 | 48857 | 48818 | 4.00E-12 |
| *Lycoperdon pratense* | 97.5% | 40 | 1 | 0 | 48818 | 48857 | 17533 | 17494 | 4.00E-12 |
| *Lycoperdon pratense* | 95.238% | 42 | 2 | 0 | 29724 | 29765 | 52928 | 52969 | 1.00E-11 |
| *Lycoperdon pratense* | 95.238% | 42 | 2 | 0 | 52928 | 52969 | 29724 | 29765 | 1.00E-11 |
| *Lycoperdon pratense* | 100% | 36 | 0 | 0 | 47742 | 47777 | 47777 | 47742 | 1.00E-11 |

| **Table S5 Tandem repeats detected in the mitogenomes of Lycoperdaceae using the online program Tandem Repeats Finder with default parameters** | | | | | | | | | |
| --- | --- | --- | --- | --- | --- | --- | --- | --- | --- |
| Species | Indices | Period Size | Copy Number | Consensus Size | Percent Matches | Percent Indels | Score | Entropy(0-2) |  |
| *Calvatia boninensis* | 3251--3280 | 14 | 2.1 | 14 | 100 | 0 | 60 | 1.9 | AGTAACCTTTGGTT |
| *Calvatia boninensis* | 14836--14864 | 11 | 2.7 | 11 | 94 | 5 | 51 | 0.58 | AAAACAAACAA |
| *Calvatia boninensis* | 31618--31643 | 13 | 2 | 13 | 100 | 0 | 52 | 0.62 | TTTTGTTTTTTTG |
| *Calvatia boninensis* | 44895--44946 | 20 | 2.6 | 20 | 93 | 0 | 86 | 1.65 | TATTTTCCAAATGATTCAAA |
| *Calvatia boninensis* | 44895--44975 | 20 | 4 | 21 | 87 | 3 | 69 | 1.75 | TATTTTCGAAATAACTCGAAA |
| *Calvatia boninensis* | 52347--52379 | 10 | 3.3 | 10 | 87 | 12 | 50 | 1.5 | ATTTATCTAA |
| *Calvatia boninensis* | 59460--59503 | 21 | 2 | 23 | 82 | 8 | 56 | 1.3 | TAATGTTTATAAATCTATGTATT |
| *Calvatia boninensis* | 60155--60183 | 14 | 2.1 | 14 | 100 | 0 | 58 | 1.89 | AGTAACCTTTGGTT |
| *Calvatia boninensis* | 60307--60354 | 25 | 1.9 | 25 | 100 | 0 | 96 | 1.89 | AAAGAGTAGTAACCTTTGGTTAGTC |
| *Calvatia caatinguensis* | 3245--3274 | 14 | 2.1 | 14 | 100 | 0 | 60 | 1.9 | AGTAACCTTTGGTT |
| *Calvatia caatinguensis* | 5768--5810 | 23 | 2 | 23 | 86 | 9 | 63 | 1.91 | ATAAAGCATGGATAGCATTCATC |
| *Calvatia caatinguensis* | 16130--16160 | 14 | 2.2 | 14 | 100 | 0 | 62 | 1.88 | TAGTAACCTTTGGT |
| *Calvatia caatinguensis* | 34348--34374 | 14 | 1.9 | 14 | 100 | 0 | 54 | 1.9 | AGTAACCTTTGGT |
| *Calvatia caatinguensis* | 41925--41976 | 20 | 2.6 | 20 | 93 | 0 | 86 | 1.65 | TATTTTCCAAATGATTCAAA |
| *Calvatia caatinguensis* | 41925--42005 | 20 | 4 | 21 | 87 | 3 | 69 | 1.75 | TATTTTCGAAATAACTCGAAA |
| *Calvatia caatinguensis* | 55030--55073 | 21 | 2 | 23 | 82 | 8 | 56 | 1.3 | TAATGTTTATAAATCTATGTATT |
| *Calvatia caatinguensis* | 55719--55747 | 14 | 2.1 | 14 | 100 | 0 | 58 | 1.89 | AGTAACCTTTGGTT |
| *Calvatia caatinguensis* | 55871--55918 | 25 | 1.9 | 25 | 100 | 0 | 96 | 1.89 | AAAGAGTAGTAACCTTTGGTTAGTC |
| *Lycoperdon perlatum* | 13278--13321 | 19 | 2.2 | 20 | 88 | 4 | 63 | 1.38 | AATTATAACAACTACAAATT |
| *Lycoperdon perlatum* | 32751--32785 | 14 | 2.5 | 14 | 90 | 0 | 52 | 1.98 | CAGCATTCTAGCAG |
| *Lycoperdon perlatum* | 34679--34715 | 15 | 2.5 | 15 | 100 | 0 | 74 | 1.28 | AAAATATTAAAGATG |
| *Lycoperdon perlatum* | 35013--35040 | 8 | 3.5 | 8 | 100 | 0 | 56 | 1.56 | GCTGGCCT |
| *Lycoperdon perlatum* | 37219--37287 | 31 | 2.2 | 32 | 89 | 5 | 113 | 1.99 | AGCTAGAATGCTGCCGTAGGCTTTACCTAGAG |
| *Lycoperdon perlatum* | 50764--50823 | 12 | 5.5 | 12 | 66 | 22 | 51 | 1.68 | GAATTATTTGAA |
| *Lycoperdon perlatum* | 50758--50853 | 21 | 4.6 | 21 | 96 | 0 | 174 | 1.79 | TTACCTGAATTATTTGAAGAA |
| *Lycoperdon pratense* | 5901--5926 | 13 | 2 | 13 | 100 | 0 | 52 | 1.95 | AGGCTTGCAGCCA |
| *Lycoperdon pratense* | 18974--19006 | 15 | 2.2 | 15 | 100 | 0 | 66 | 1.94 | CTAGGCTAGCGAAAG |
| *Lycoperdon pratense* | 25067--25115 | 15 | 3.3 | 15 | 94 | 0 | 89 | 1.37 | AGATGATAAAAATAA |
| *Lycoperdon pratense* | 25118--25147 | 15 | 2 | 15 | 93 | 0 | 51 | 1.69 | AGATAAGAAATCATC |
| *Lycoperdon pratense* | 40384--40419 | 17 | 2.1 | 17 | 94 | 0 | 63 | 1.33 | AAATTGAAATAATCTAA |

| **Table S6 Distribution of repeat loci in the mitogenomes of Lycoperdaceae searched by REPuter** | | | | | | |
| --- | --- | --- | --- | --- | --- | --- |
| **Species** | **1st repeat length** | **Starting position** | **Match direction** | **2nd repeat length** | **Starting position** | **E-value** |
| *Calvatia boninensis* | 23 | 11200 | F | 23 | 28101 | 1.54E-05 |
| *Calvatia boninensis* | 23 | 13469 | F | 23 | 37479 | 1.54E-05 |
| *Calvatia boninensis* | 23 | 24729 | F | 23 | 32356 | 1.54E-05 |
| *Calvatia boninensis* | 23 | 60306 | F | 23 | 60331 | 1.54E-05 |
| *Calvatia boninensis* | 24 | 1619 | F | 24 | 9448 | 3.86E-06 |
| *Calvatia boninensis* | 24 | 18779 | F | 24 | 56051 | 3.86E-06 |
| *Calvatia boninensis* | 24 | 37447 | F | 24 | 60148 | 3.86E-06 |
| *Calvatia boninensis* | 25 | 8149 | F | 25 | 14078 | 9.65E-07 |
| *Calvatia boninensis* | 25 | 32060 | F | 25 | 34530 | 9.65E-07 |
| *Calvatia boninensis* | 26 | 11549 | F | 26 | 57342 | 2.41E-07 |
| *Calvatia boninensis* | 27 | 28835 | F | 27 | 61956 | 6.03E-08 |
| *Calvatia boninensis* | 29 | 18796 | F | 29 | 37832 | 3.77E-09 |
| *Calvatia boninensis* | 30 | 1723 | F | 30 | 9522 | 9.42E-10 |
| *Calvatia boninensis* | 31 | 3248 | F | 31 | 60152 | 2.36E-10 |
| *Calvatia boninensis* | 34 | 55786 | F | 34 | 57727 | 3.68E-12 |
| *Calvatia boninensis* | 35 | 57665 | F | 35 | 58863 | 9.20E-13 |
| *Calvatia boninensis* | 42 | 26250 | F | 42 | 32161 | 5.62E-17 |
| *Calvatia boninensis* | 44 | 158 | F | 44 | 1531 | 3.51E-18 |
| *Calvatia boninensis* | 46 | 24692 | F | 46 | 26490 | 2.19E-19 |
| *Calvatia boninensis* | 48 | 27124 | F | 48 | 44431 | 1.37E-20 |
| *Calvatia boninensis* | 53 | 24685 | F | 53 | 32307 | 1.34E-23 |
| *Calvatia boninensis* | 61 | 26490 | F | 61 | 32314 | 2.04E-28 |
| *Calvatia boninensis* | 77 | 5153 | F | 77 | 59231 | 4.76E-38 |
| *Calvatia boninensis* | 138 | 57810 | F | 138 | 58930 | 8.95E-75 |
| *Calvatia boninensis* | 23 | 61719 | R | 23 | 61719 | 1.54E-05 |
| *Calvatia boninensis* | 29 | 14837 | R | 29 | 14837 | 3.77E-09 |
| *Calvatia boninensis* | 23 | 3245 | P | 23 | 58730 | 1.54E-05 |
| *Calvatia boninensis* | 23 | 11310 | P | 23 | 37477 | 1.54E-05 |
| *Calvatia boninensis* | 23 | 56230 | P | 23 | 60311 | 1.54E-05 |
| *Calvatia boninensis* | 24 | 5192 | P | 24 | 32762 | 3.86E-06 |
| *Calvatia boninensis* | 24 | 6080 | P | 24 | 18770 | 3.86E-06 |
| *Calvatia boninensis* | 24 | 11195 | P | 24 | 11647 | 3.86E-06 |
| *Calvatia boninensis* | 24 | 32762 | P | 24 | 59270 | 3.86E-06 |
| *Calvatia boninensis* | 24 | 35231 | P | 24 | 36486 | 3.86E-06 |
| *Calvatia boninensis* | 24 | 60169 | P | 24 | 60489 | 3.86E-06 |
| *Calvatia boninensis* | 25 | 22708 | P | 25 | 37482 | 9.65E-07 |
| *Calvatia boninensis* | 26 | 28622 | P | 26 | 28622 | 2.41E-07 |
| *Calvatia boninensis* | 30 | 61946 | P | 30 | 61946 | 9.42E-10 |
| *Calvatia boninensis* | 33 | 37451 | P | 33 | 58717 | 1.47E-11 |
| *Calvatia boninensis* | 35 | 56698 | P | 35 | 57394 | 9.20E-13 |
| *Calvatia boninensis* | 36 | 57581 | P | 36 | 60967 | 2.30E-13 |
| *Calvatia boninensis* | 38 | 9478 | P | 38 | 9478 | 1.44E-14 |
| *Calvatia boninensis* | 38 | 61463 | P | 38 | 61463 | 1.44E-14 |
| *Calvatia boninensis* | 39 | 22690 | P | 39 | 28105 | 3.59E-15 |
| *Calvatia caatinguensis* | 22 | 180 | F | 22 | 1553 | 5.33E-05 |
| *Calvatia caatinguensis* | 22 | 13083 | F | 22 | 26627 | 5.33E-05 |
| *Calvatia caatinguensis* | 22 | 14506 | F | 22 | 56536 | 5.33E-05 |
| *Calvatia caatinguensis* | 23 | 55870 | F | 23 | 55895 | 1.33E-05 |
| *Calvatia caatinguensis* | 24 | 1619 | F | 24 | 9053 | 3.33E-06 |
| *Calvatia caatinguensis* | 24 | 18390 | F | 24 | 51629 | 3.33E-06 |
| *Calvatia caatinguensis* | 24 | 21004 | F | 24 | 57549 | 3.33E-06 |
| *Calvatia caatinguensis* | 24 | 34820 | F | 24 | 51629 | 3.33E-06 |
| *Calvatia caatinguensis* | 26 | 16891 | F | 26 | 31969 | 2.08E-07 |
| *Calvatia caatinguensis* | 26 | 23773 | F | 26 | 25183 | 2.08E-07 |
| *Calvatia caatinguensis* | 27 | 14087 | F | 27 | 53159 | 5.20E-08 |
| *Calvatia caatinguensis* | 27 | 16130 | F | 27 | 34347 | 5.20E-08 |
| *Calvatia caatinguensis* | 27 | 30508 | F | 27 | 33762 | 5.20E-08 |
| *Calvatia caatinguensis* | 29 | 3242 | F | 29 | 34345 | 3.25E-09 |
| *Calvatia caatinguensis* | 29 | 16130 | F | 29 | 55718 | 3.25E-09 |
| *Calvatia caatinguensis* | 30 | 1723 | F | 30 | 9127 | 8.13E-10 |
| *Calvatia caatinguensis* | 30 | 10796 | F | 30 | 27028 | 8.13E-10 |
| *Calvatia caatinguensis* | 31 | 3242 | F | 31 | 55716 | 2.03E-10 |
| *Calvatia caatinguensis* | 31 | 3244 | F | 31 | 16130 | 2.03E-10 |
| *Calvatia caatinguensis* | 33 | 34341 | F | 33 | 55712 | 1.27E-11 |
| *Calvatia caatinguensis* | 34 | 51364 | F | 34 | 53297 | 3.18E-12 |
| *Calvatia caatinguensis* | 35 | 53235 | F | 35 | 54433 | 7.94E-13 |
| *Calvatia caatinguensis* | 48 | 26060 | F | 48 | 41480 | 1.18E-20 |
| *Calvatia caatinguensis* | 56 | 18380 | F | 56 | 34810 | 1.81E-25 |
| *Calvatia caatinguensis* | 67 | 23896 | F | 67 | 25426 | 4.31E-32 |
| *Calvatia caatinguensis* | 138 | 53380 | F | 138 | 54500 | 7.72E-75 |
| *Calvatia caatinguensis* | 23 | 57283 | R | 23 | 57283 | 1.33E-05 |
| *Calvatia caatinguensis* | 23 | 3239 | P | 23 | 54300 | 1.33E-05 |
| *Calvatia caatinguensis* | 23 | 51808 | P | 23 | 55875 | 1.33E-05 |
| *Calvatia caatinguensis* | 24 | 10798 | P | 24 | 11226 | 3.33E-06 |
| *Calvatia caatinguensis* | 24 | 11226 | P | 24 | 27030 | 3.33E-06 |
| *Calvatia caatinguensis* | 24 | 13138 | P | 24 | 13138 | 3.33E-06 |
| *Calvatia caatinguensis* | 24 | 14716 | P | 24 | 14716 | 3.33E-06 |
| *Calvatia caatinguensis* | 24 | 33531 | P | 24 | 34361 | 3.33E-06 |
| *Calvatia caatinguensis* | 24 | 40410 | P | 24 | 54450 | 3.33E-06 |
| *Calvatia caatinguensis* | 24 | 55733 | P | 24 | 56053 | 3.33E-06 |
| *Calvatia caatinguensis* | 27 | 14087 | P | 27 | 56532 | 5.20E-08 |
| *Calvatia caatinguensis* | 30 | 57510 | P | 30 | 57510 | 8.13E-10 |
| *Calvatia caatinguensis* | 35 | 52276 | P | 35 | 52972 | 7.94E-13 |
| *Calvatia caatinguensis* | 37 | 53150 | P | 37 | 56531 | 4.96E-14 |
| *Calvatia caatinguensis* | 38 | 57027 | P | 38 | 57027 | 1.24E-14 |
| *Lycoperdon perlatum* | 23 | 8063 | F | 23 | 26361 | 1.11E-05 |
| *Lycoperdon perlatum* | 23 | 9773 | F | 23 | 16153 | 1.11E-05 |
| *Lycoperdon perlatum* | 24 | 8040 | F | 24 | 46838 | 2.77E-06 |
| *Lycoperdon perlatum* | 24 | 8065 | F | 24 | 25445 | 2.77E-06 |
| *Lycoperdon perlatum* | 24 | 8475 | F | 24 | 10953 | 2.77E-06 |
| *Lycoperdon perlatum* | 24 | 10953 | F | 24 | 42666 | 2.77E-06 |
| *Lycoperdon perlatum* | 24 | 11922 | F | 24 | 26789 | 2.77E-06 |
| *Lycoperdon perlatum* | 24 | 40164 | F | 24 | 40315 | 2.77E-06 |
| *Lycoperdon perlatum* | 24 | 50812 | F | 24 | 50833 | 2.77E-06 |
| *Lycoperdon perlatum* | 25 | 8134 | F | 25 | 46886 | 6.94E-07 |
| *Lycoperdon perlatum* | 25 | 50757 | F | 25 | 50778 | 6.94E-07 |
| *Lycoperdon perlatum* | 26 | 8474 | F | 26 | 42665 | 1.73E-07 |
| *Lycoperdon perlatum* | 27 | 9541 | F | 27 | 35774 | 4.34E-08 |
| *Lycoperdon perlatum* | 27 | 40597 | F | 27 | 42730 | 4.34E-08 |
| *Lycoperdon perlatum* | 28 | 8439 | F | 28 | 13322 | 1.08E-08 |
| *Lycoperdon perlatum* | 28 | 32847 | F | 28 | 35525 | 1.08E-08 |
| *Lycoperdon perlatum* | 28 | 50762 | F | 28 | 50804 | 1.08E-08 |
| *Lycoperdon perlatum* | 30 | 18621 | F | 30 | 23865 | 6.77E-10 |
| *Lycoperdon perlatum* | 32 | 9766 | F | 32 | 13502 | 4.23E-11 |
| *Lycoperdon perlatum* | 32 | 24210 | F | 32 | 46886 | 4.23E-11 |
| *Lycoperdon perlatum* | 33 | 8126 | F | 33 | 24202 | 1.06E-11 |
| *Lycoperdon perlatum* | 33 | 9640 | F | 33 | 35873 | 1.06E-11 |
| *Lycoperdon perlatum* | 34 | 44121 | F | 34 | 44909 | 2.65E-12 |
| *Lycoperdon perlatum* | 35 | 22142 | F | 35 | 26924 | 6.61E-13 |
| *Lycoperdon perlatum* | 37 | 9409 | F | 37 | 35329 | 4.13E-14 |
| *Lycoperdon perlatum* | 38 | 13446 | F | 38 | 27274 | 1.03E-14 |
| *Lycoperdon perlatum* | 43 | 8564 | F | 43 | 13405 | 1.01E-17 |
| *Lycoperdon perlatum* | 46 | 8517 | F | 46 | 13358 | 1.58E-19 |
| *Lycoperdon perlatum* | 47 | 8560 | F | 47 | 27228 | 3.94E-20 |
| *Lycoperdon perlatum* | 48 | 13405 | F | 48 | 27232 | 9.86E-21 |
| *Lycoperdon perlatum* | 56 | 9569 | F | 56 | 35802 | 1.50E-25 |
| *Lycoperdon perlatum* | 23 | 2633 | P | 23 | 30075 | 1.11E-05 |
| *Lycoperdon perlatum* | 23 | 9958 | P | 23 | 47549 | 1.11E-05 |
| *Lycoperdon perlatum* | 23 | 9958 | P | 23 | 52331 | 1.11E-05 |
| *Lycoperdon perlatum* | 24 | 15472 | P | 24 | 16385 | 2.77E-06 |
| *Lycoperdon perlatum* | 25 | 2635 | P | 25 | 13298 | 6.94E-07 |
| *Lycoperdon perlatum* | 26 | 12798 | P | 26 | 47983 | 1.73E-07 |
| *Lycoperdon perlatum* | 26 | 49561 | P | 26 | 49561 | 1.73E-07 |
| *Lycoperdon perlatum* | 27 | 2635 | P | 27 | 15429 | 4.34E-08 |
| *Lycoperdon perlatum* | 28 | 6182 | P | 28 | 6182 | 1.08E-08 |
| *Lycoperdon perlatum* | 30 | 47780 | P | 30 | 47780 | 6.77E-10 |
| *Lycoperdon perlatum* | 31 | 33718 | P | 31 | 51710 | 1.69E-10 |
| *Lycoperdon perlatum* | 31 | 38066 | P | 31 | 49272 | 1.69E-10 |
| *Lycoperdon perlatum* | 32 | 44014 | P | 32 | 52468 | 4.23E-11 |
| *Lycoperdon perlatum* | 32 | 47539 | P | 32 | 48625 | 4.23E-11 |
| *Lycoperdon perlatum* | 32 | 48625 | P | 32 | 52321 | 4.23E-11 |
| *Lycoperdon perlatum* | 33 | 49260 | P | 33 | 52465 | 1.06E-11 |
| *Lycoperdon perlatum* | 34 | 17610 | P | 34 | 38283 | 2.65E-12 |
| *Lycoperdon perlatum* | 36 | 6004 | P | 36 | 6004 | 1.65E-13 |
| *Lycoperdon perlatum* | 61 | 16820 | P | 61 | 38258 | 1.47E-28 |
| *Lycoperdon pratense* | 23 | 2490 | F | 23 | 29061 | 1.31E-05 |
| *Lycoperdon pratense* | 23 | 2914 | F | 23 | 46091 | 1.31E-05 |
| *Lycoperdon pratense* | 23 | 5829 | F | 23 | 15172 | 1.31E-05 |
| *Lycoperdon pratense* | 24 | 12421 | F | 24 | 35289 | 3.29E-06 |
| *Lycoperdon pratense* | 24 | 24224 | F | 24 | 47518 | 3.29E-06 |
| *Lycoperdon pratense* | 25 | 28628 | F | 25 | 47658 | 8.22E-07 |
| *Lycoperdon pratense* | 27 | 16225 | F | 27 | 18735 | 5.14E-08 |
| *Lycoperdon pratense* | 27 | 17188 | F | 27 | 42184 | 5.14E-08 |
| *Lycoperdon pratense* | 27 | 18786 | F | 27 | 42184 | 5.14E-08 |
| *Lycoperdon pratense* | 29 | 22584 | F | 29 | 28709 | 3.21E-09 |
| *Lycoperdon pratense* | 29 | 27799 | F | 29 | 36224 | 3.21E-09 |
| *Lycoperdon pratense* | 30 | 12407 | F | 30 | 12574 | 8.03E-10 |
| *Lycoperdon pratense* | 30 | 39292 | F | 30 | 40704 | 8.03E-10 |
| *Lycoperdon pratense* | 32 | 18678 | F | 32 | 52994 | 5.02E-11 |
| *Lycoperdon pratense* | 34 | 29723 | F | 34 | 52927 | 3.13E-12 |
| *Lycoperdon pratense* | 35 | 9003 | F | 35 | 12330 | 7.84E-13 |
| *Lycoperdon pratense* | 37 | 17179 | F | 37 | 18777 | 4.90E-14 |
| *Lycoperdon pratense* | 39 | 17332 | F | 39 | 18570 | 3.06E-15 |
| *Lycoperdon pratense* | 39 | 21688 | F | 39 | 48983 | 3.06E-15 |
| *Lycoperdon pratense* | 40 | 13126 | F | 40 | 15003 | 7.65E-16 |
| *Lycoperdon pratense* | 49 | 29907 | F | 49 | 31017 | 2.92E-21 |
| *Lycoperdon pratense* | 51 | 2718 | F | 51 | 50869 | 1.82E-22 |
| *Lycoperdon pratense* | 53 | 15966 | F | 53 | 16113 | 1.14E-23 |
| *Lycoperdon pratense* | 23 | 4917 | R | 23 | 4917 | 1.31E-05 |
| *Lycoperdon pratense* | 23 | 5420 | P | 23 | 29089 | 1.31E-05 |
| *Lycoperdon pratense* | 23 | 6198 | P | 23 | 26716 | 1.31E-05 |
| *Lycoperdon pratense* | 23 | 9003 | P | 23 | 49189 | 1.31E-05 |
| *Lycoperdon pratense* | 23 | 12224 | P | 23 | 18740 | 1.31E-05 |
| *Lycoperdon pratense* | 24 | 2914 | P | 24 | 47500 | 3.29E-06 |
| *Lycoperdon pratense* | 24 | 8833 | P | 24 | 9324 | 3.29E-06 |
| *Lycoperdon pratense* | 24 | 46090 | P | 24 | 47501 | 3.29E-06 |
| *Lycoperdon pratense* | 26 | 26711 | P | 26 | 33732 | 2.05E-07 |
| *Lycoperdon pratense* | 26 | 28666 | P | 26 | 35314 | 2.05E-07 |
| *Lycoperdon pratense* | 27 | 12326 | P | 27 | 49189 | 5.14E-08 |
| *Lycoperdon pratense* | 28 | 2444 | P | 28 | 8997 | 1.28E-08 |
| *Lycoperdon pratense* | 28 | 41732 | P | 28 | 48793 | 1.28E-08 |
| *Lycoperdon pratense* | 29 | 24205 | P | 29 | 24705 | 3.21E-09 |
| *Lycoperdon pratense* | 31 | 13531 | P | 31 | 18679 | 2.01E-10 |
| *Lycoperdon pratense* | 31 | 13531 | P | 31 | 52995 | 2.01E-10 |
| *Lycoperdon pratense* | 32 | 36092 | P | 32 | 36092 | 5.02E-11 |
| *Lycoperdon pratense* | 35 | 17493 | P | 35 | 48822 | 7.84E-13 |
| *Lycoperdon pratense* | 36 | 29471 | P | 36 | 40421 | 1.96E-13 |
| *Lycoperdon pratense* | 36 | 47741 | P | 36 | 47741 | 1.96E-13 |
| *Lycoperdon pratense* | 40 | 13126 | P | 40 | 15966 | 7.65E-16 |
| *Lycoperdon pratense* | 42 | 2727 | P | 42 | 48066 | 4.78E-17 |
| *Lycoperdon pratense* | 44 | 13126 | P | 44 | 16109 | 2.99E-18 |
| *Lycoperdon pratense* | 47 | 5825 | P | 47 | 52552 | 4.67E-20 |
| *Lycoperdon pratense* | 47 | 14996 | P | 47 | 16113 | 4.67E-20 |
| *Lycoperdon pratense* | 58 | 48050 | P | 58 | 50878 | 1.11E-26 |
| *Lycoperdon pratense* | 62 | 14996 | P | 62 | 15951 | 4.35E-29 |

| **Table S7 Species information used for phylogenetic analysis in this study** | | | | | |
| --- | --- | --- | --- | --- | --- |
| **ID** | **Species** | **GenBank accession number** | **Family** | **genus** | **Source** |
| AB | *Agaricus bitorquis* | NC077556 | Agaricaceae | *Agaricus* | Ferandon et al. |
| CC | *Coprinus comatus* | NC059951 |  | *Coprinus* | Unpublished |
| MF | *Macrolepiota fuliginosa* | NC045202 |  | *Macrolepiota* | Unpublished |
| LN | *Leucoagaricus naucinus* | [MZ352929](https://doi.org/10.1080/23802359.2021.1970643) |  | *Leucoagaricus* | Li et al. |
| AA | *Agrocybe aegerita* | [NC057071](https://doi.org/10.1016/j.csbj.2020.08.022) | Bolbitiaceae | *Cyclocybe* | Liu et al. |
| AB | *Amanita basii* | NC045195 | Amanitaceae | *Amanita* | Li et al. |
| AB | *Amanita bisporigera* | NC045196 |  |  | Li et al. |
| AB | *Amanita brunnescens* | NC045197 |  |  | Li et al. |
| AI | *Amanita inopinata* | NC045198 |  |  | Li et al. |
| AB | *Armillaria borealis* | NC042230 | Physalacriaceae | *Armillaria* | Anna et al. |
| AS | *Armillaria sinapina* | NC042229 |  |  | Anna et al. |
| AS | *Armillaria solidipes* | NC042231 |  |  | Anna et al. |
| DT | *Desarmillaria tabescens* | NC042769 |  | *Desarmillaria* | Lee et al. |
| BH | *Butyriboletus hainanensis* | NC082970 | Boletaceae | *Rufoboletus* | Unpublished |
| BR | *Butyriboletus roseoflavus* | MZ202357 |  | *Butyriboletus* | Li et al. |
| AR | *Aureoboletus raphanaceus* | NC079662 |  | *Aureoboletus* | Unpublished |
| XI | *Xerocomus impolitus* | NC056808 |  | *Xerocomus* | Unpublished |
| LM | *Lanmaoa macrocarpa* | NC080885 |  | *Lanmaoa* | Zheng et al. |
| PR | *Pulveroboletus ravenelii* | NC061666 |  | *Pulveroboletus* | Cho et al. |
| TP | *Tylopilus plumbeoviolaceoides* | NC056835 |  | *Tylopilus* | Shi et al. |
| CR | *Chroogomphus rutilus* | MZ151416 | Gomphidiaceae | *Chroogomphus* | Fu et al. |
| CF | *Clavaria fumosa* | NC056336 | Clavariaceae | *Clavaria* | Wang et al. |
| C sp. | *Clavulina sp.* | MT649302 | Hydnaceae | *Clavulina* | Tan et al. |
| CM | *Coprinellus micaceus* | NC057083 | Psathyrellaceae | *Coprinellus* | Wang et al. |
| CC | *Coprinopsis cinerea* | AACS02000068 |  |  | Jason et al. |
| CP | *Cyathus pallidus* | NC079580 | Nidulariaceae | *Cyathus* | Unpublished |
| CS | *Cyathus striatus* | NC072522 |  |  | Unpublished |
| GJ | *Gymnopilus junonius* | NC057300 | Hymenogastraceae | *Gymnopilus* | Cho et al. |
| HR | *Hygrophorus russula* | NC039589 | Hygrophoraceae | *Hygrophorus* | Li et al. |
| LA | *Laccaria amethystina* | NC042772 | Hydnangiaceae | *Laccaria* | Li et al. |
| LB | *Laccaria bicolor* | NC042773 |  |  | Li et al. |
| LE | *Lentinula edodes* | NC018365 | Omphalotaceae | *Lentinula* | Yasumasa et al. |
| MS | *Marasmiellus scandens* | MZ615350 |  | *Marasmiellus* | Shahin S, A. et al. |
| OJ | *Omphalotus japonicus* | NC049125 |  | *Omphalotus* | Unpublished |
| MC | *Marasmius crinis-equi* | MZ615351 | Marasmiaceae | *Marasmius* | Shahin S, A. et al. |
| MT | *Marasmius tenuissimus* | MZ615345 |  |  | Shahin S, A. et al. |
| MP | *Moniliophthora perniciosa* | NC005927 |  | *Moniliophthora* | Eduardo et al. |
| MR | *Moniliophthora roreri* | NC015400 |  | *Moniliophthora* | Unpublished |
| PP | *Paramarasmius palmivorus* | MZ615352 |  | *Paramarasmius* | Shahin S, A. et al. |
| PI | *Paxillus involutus* | NC045203 | Paxillaceae | *Paxillus* | Li et al. |
| PR | *Paxillus rubicundulus* | NC045204 |  |  | Li et al. |
| PP | *Phlebopus portentosus* | MK571437 | Boletinellaceae | *Phlebopus* | Jiang et al. |
| PM | *Pisolithus microcarpus* | NC054201 | Pisolithaceae | *Pisolithus* | Wu et al. |
| PT | *Pisolithus tinctorius* | NC054202 |  |  | Wu et al. |
| PC | *Pleurotus citrinopileatus* | NC036998 | Pleurotaceae | *Pleurotus* | Li et al. |
| PC | *Pleurotus cornucopiae* | NC038091 |  |  | Xu et al. |
| PE | *Pleurotus eryngii* | NC033533 |  |  | Yang et al. |
| RR | *Ramaria rubella* | NC068232 | Gomphaceae | *Ramaria* | Li et al. |
| RS | *Rhizopogon salebrosus* | NC042698 | Rhizopogonaceae | *Rhizopogon* | Li et al. |
| RV | *Rhizopogon vinicolor* | NC042699 |  |  | Li et al. |
| SC | *Schizophyllum commune* | NC003049 | Schizophyllaceae | *Schizophyllum* | Lise et al. |
| SR | *Stropharia rugosoannulata* | AP019006 | Strophariaceae | *Stropharia* | Tomohiro et al. |
| TR | *Tephrocybe rancida* | NC039442 | Lyophyllaceae | *Tephrocybe* | Mathijs et al. |
| T sp. | *Termitomyces sp.* | MH725795 |  | *Termitomyces* | Mathijs et al. |
| LD | *Lyophyllum decastes* | NC038223 |  | *Lyophyllum* | Mathijs et al. |
| LS | *Lyophyllum shimeji* | NC038224 |  |  | Mathijs et al. |
| TC | *Tricholomella constricta* | NC039443 |  | *Tricholomella* | Mathijs et al. |
| AP | *Asterophora parasitica* | NC039439 |  | *Asterophora* | Mathijs et al. |
| HM | *Hypsizygus marmoreus* | NC042219 |  | *Hypsizygus* | Wang et al. |
| BZ | *Blastosporella zonata* | NC039440 |  | *Blastosporella* | Mathijs et al. |
| TF | *Tricholoma flavovirens* | NC046501 | Tricholomatineae | *Tricholoma* | Huang et al. |
| TL | *Tricholoma lobayense* | NC064402 |  |  | Unpublished |
| LS | *Lepista sordida* | LC565488 |  | *Collybia* | Choi et al. |
| VV | *Volvariella volvacea* | NC044971 | Pluteaceae | *Volvariella* | Unpublished |
| M1126 | *Lycoperdon perlatum* | PP690777 | Lycoperdaceae | *Lycoperdon* | This study |
| M817 | *Lycoperdon pratense* | PP697973 |  |  | This study |
| M842 | *Calvatia boninensis* | PP670003 |  | *Calvatia* | This study |
| M867 | *Calvatia caatinguensis* | PP840861 |  |  | This study |

**References**

Anna I., K., Yuliya A., P., Evgeniy P., S., Vladislav V., B., Natalya V., O., Igor N., P., Vadim V., S., Dmitry A., K., James B., A., Konstantin V., K. 2019. Mobile genetic elements explain size variation in the mitochondrial genomes of four closely-related *Armillaria* species, BMC Genomics, 20.1: 351.

Cong, Peng., Zhijie, Bao., Wenying, Tu., Lijiao, Li., Qiang, Li. 2021. The first complete mitochondrial genome of mushroom *Leucoagaricus naucinus* (Agaricaceae, Agaricales) and insights into its phylogeny., Mitochondrial DNA B, 6.10: 2803-2805.

Eduardo F., F., Ricardo A., T., Eduardo D., A., Francisco J., M., Hugo, S., Nicolas, C., Aristóteles, G., Carolina, C., Marcelo F., C., Naiara, S., Daniela P.T., T., Johana, R., Luciano, D., Dirce M., C., Ana M., A., Sérgio F., R., Ana C., D., Karina, G., Marilda S., G., José P. Moura, N., Luciana V., B., Lyndel W., M., Júlio C.M., C., Gonçalo A.G., P. 2008. The mitochondrial genome of the phytopathogenic basidiomycete *Moniliophthora perniciosa* is 109kb in size and contains a stable integrated plasmid, Mycol. Res. 112.10: 1136-1152.

Ferandon C, Xu J, Barroso G. The 135 Kbp Mitochondrial Genome of *Agaricus Bisporus* is the Largest Known Eukaryotic Reservoir of Group I Introns and Plasmid-Related Sequences. Fungal Genetics and Biology. 2013;55:85-91.

Gang, Wang., Jingxian, Lin., Yang, Shi., Xiaoguang, Chang., Yuanyuan, Wang., Lin, Guo., Wenhui, Wang., Meijie, Dou., Youjin, Deng., Ray, Ming., Jisen, Zhang. 2019. Mitochondrial genome in *Hypsizygus marmoreus* and its evolution in Dikarya, BMC Genomics, 20.1: 765.

Hwa-Yong, Lee., Suyun, M., Chang-Duck, Koo., Jong-Wook, Chung., Hojin, Ryu. 2019. The complete mitochondrial genome of the edible and phytopathogenic fungus *Desarmillaria tabescens*, Mitochondrial DNA B, 4.1: 33.0-34.

Jason E., S., Sarah K., W., Dag, A., Chun Hang, Au., Bruce W., B., Mark, B., Claire, B., Bjorn, C., Lorna A., C., C. K., C., Jixin, Deng., Fred S., D., David C., F., Mark L., F., Allen C., G., Jonathan, G., Roderic, G., Patrick J., H., James B., H., Ashleigh, H., Timothy Y., J., Takashi, K., Sreedhar, K., Chinnapa, K., Ursula, K., Doris, K., H. S., K., Alexandre, L., Weixi, Li., Walt W., L., Li-Jun, M., Aaron J., M., Gerard, M., Francis, M., Hajime, M., Donald O., N., Heather, P., Marilee A., R., Cathy J., R., Bruce A., R., Narmada, S., Mario, S., Vardges, T., Anders, T., Rajesh, V., Todd J., V., Qiandong, Zeng., Miriam E., Z., Patricia J., P. 2010. Insights Into Evolution Of Multicellular Fungi From The Assembled Chromosomes Of The Mushroom *Coprinopsis Cinerea* (*Coprinus Cinereus*), Proceedings of the National Academy of Sciences of the United States of America, 107.26: 11889-11894.

Jia, Fu., Wenying, Tu., Zhijie, Bao., Lijiao, Li., Qiang, Li. 2021. The First Complete Mitochondrial Genome Of Edible And Medicinal Fungus *Chroogomphus Rutilus* (Gomphidiaceae, Boletales) And Insights Into Its Phylogeny, Mitochondrial DNA B, 6.8: 2355-2357.

Jae-Hoon, Choi., Tomohiro, S., Akiko, O., Mihaya, K., Yuki, T., Toshiyuki, S., Hirokazu, K., Hideo, D. 2022. The complete mitochondrial genome sequence of the fairy ring-forming fungus *Lepista sordida*, Mitochondrial DNA B, 7.4: 712-714.

Lili, Jiang., Dan, Yang., Yang, Cao., Pengfei, Wang., Yunrun, Zhang., Ke-Qin, Zhang., Jianping, Xu., Ying, Zhang. 2017. The complete mitochondrial genome of the edible Basidiomycete mushroom *Phlebopus Portentosus*., Mitochondrial DNA B, 2.2: 696.0-697.

Liming, Xu., Damien Daniel, H., Guofeng, Jiang. 2018. The complete mitochondrial genome of the Basidiomycete fungus *Pleurotus cornucopiae* (Paulet) Rolland., Mitochondrial DNA B, 3.1: 73-75.

Lise, F., Jana, U., Zhang, W., Volker A R, H., B Franz, L. 2002. Hyaloraphidium Curvatum: A Linear Mitochondrial Genome, Trna Editing, And An Evolutionary Link To Lower Fungi, Mol. Biol. Evol. 19.3: 310-319.

Mathijs, N., Lennart J J van de, P., Freek T, B., Bas J, Z., Duur K, A. 2019. Enrichment of G4DNA and a large inverted repeat coincide in the mitochondrial genomes of *Termitomyces*., Genome. Biol. Evol. 11.7.0: 1857.0-1869.

Maoling, Tan., Gang, Zhao. 2020. Characterization And Phylogenetic Analysis Of The Complete Mitochondrial Genome Of *Clavulina* Sp. (Cantharellales: Clavulinaceae), Mitochondrial DNA B, 5.3: 2944-2945.

Peng, Wu., Tian, Yao., Yuanhang, Ren., Jinghua, Ye., Yuan, Qing., Qiang, Li., Mingying, Gui. 2021. Evolutionary Insights Into Two Widespread Ectomycorrhizal Fungi (*Pisolithus*) From Comparative Analysis of Mitochondrial Genomes., Front. Microbiol. 12: 583129.

Qiang, Li., Xiaohui, He., Yuanhang, Ren., Chuan, Xiong., Xin, Jin., Lianxin, Peng., Wenli, Huang. 2020. Comparative Mitogenome Analysis Reveals Mitochondrial Genome Differentiation in Ectomycorrhizal and Asymbiotic *Amanita* Species., Front. Microbiol. 11: 1382.

Qiang, Li., Qiangfeng, Wang., Xin, Jin., Zuqin, Chen., Chuan, Xiong., Ping, Li., Jian, Zhao., Wenli, Huang. 2018. The first complete mitochondrial genome from the family Hygrophoraceae (*Hygrophorus russula*) by next-generation sequencing and phylogenetic implications., Int. J. Biol. Macromol. 122.: 1313-1320.

Qiang, Li., Luxi, Yang., Dabing, Xiang., Yan, Wan., Qi, Wu., Wenli, Huang., Gang, Zhao. 2019. The complete mitochondrial genomes of two model ectomycorrhizal fungi (*Laccaria*): features, intron dynamics and phylogenetic implications., Int. J. Biol. Macromol. 145.: 974-984.

Qiang, Li., Qiangfeng, Wang., Xin, Jin., Zuqin, Chen., Chuan, Xiong., Ping, Li., Jian, Zhao., Wenli, Huang. 2019. Characterization and comparison of the mitochondrial genomes from two Lyophyllum fungal species and insights into phylogeny of Agaricomycetes, Int. J. Biol. Macromol. 121.: 364-372.

Qiang, Li., Yuanhang, Ren., Dabing, Xiang., Xiaodong, Shi., Jianglin, Zhao., Lianxin, Peng., Gang, Zhao. 2020. Comparative mitogenome analysis of two ectomycorrhizal fungi (*Paxillus*) reveals gene rearrangement, intron dynamics, and phylogeny of basidiomycetes., IMA fungus, 11.1: 12.

Qiang, L., Cheng, C., Chuan, X., Xin, J., Zuqin, C., Wenli, H. 2018. Comparative mitogenomics reveals large-scale gene rearrangements in the mitochondrial genome of two *Pleurotus* species, Appl. Microbiol. Biot. 102.14: 6143.0-6153.0.

Qiang, Li., Lijiao, Li., Ting, Zhang., Peng, Xiang., Qian, Wu., Wenying, Tu., Zhijie, Bao., Liang, Zou., Cheng, Chen. 2022. The first two mitochondrial genomes for the genus *Ramaria* reveal mitochondrial genome evolution of *Ramaria* and phylogeny of Basidiomycota, IMA Fungus, 13.1: 1-17.

Qiang, Li., Yuanhang, Ren., Xiaodong, Shi., Lianxin, Peng., Jianglin, Zhao., Yu, Song., Gang, Zhao. 2019. Comparative Mitochondrial Genome Analysis of Two Ectomycorrhizal Fungi ( *Rhizopogon* ) Reveals Dynamic Changes of Intron and Phylogenetic Relationships of the Subphylum Agaricomycotina., Int. J. Mol. Sci. 20.20

Ruiheng, Yang., Yan, Li., Chuanhua, Li., Jianping, Xu., Daopeng, Bao. 2016. The Complete Mitochondrial Genome Of The Basidiomycete Edible Fungus Pleurotus Eryngii, Mitochondrial DNA B, 1.1: 772-774.

Shurong, Wang., Jiangping, Zhang., Yirong, He., Mingchang, Chang., Junlong, Meng. 2021. Characterization Of The Complete Mitochondrial Genome Of *Coprinellus Micaceus*, A Wild Saprobic Mushroom In China, Mitochondrial DNA B, 6.7: 1979-1981.

Sung Eun, C., Jong Won, J., Young-Nam, K., Hyun, L., Jong-Wook, C., Seung Hwan, O., Chang Sun, K. 2021. Complete Mitochondrial Genome Sequence Of *Gymnopilus Junonius*, Mitochondrial DNA B, 6.3: 1020-1021.

Shahin S, A., Ishmael, A., Jonathan, S., Eric, K., Lyndel W, M., Bryan A, B. 2021. Mitochondrial Genomics of Six Cacao Pathogens From the Basidiomycete Family *Marasmiaceae*, Front. Microbiol. 12: 752094.

Sung-Eun, Cho., Young-Nam, Kwag., Sang-Kuk, Han., Dong-Hyeon, Lee., Chang Sun, Kim. 2022. Complete mitochondrial genome sequence of *Pulveroboletus ravenelii* (Boletales, Basidiomycota), Mitochondrial DNA B, 7.9: 1581-1582.

Tomohiro, S., Akiko, O., Jae-Hoon, C., Jing, Wu., Hirokazu, K., Hideo, D. 2019. The complete mitochondrial genome sequence of the edible mushroom *Stropharia rugosoannulata* (Strophariaceae, Basidiomycota), Mitochondrial DNA B, 4.1: 570.0-572.

Wenli, Huang., Huiyu, Feng., Wenying, Tu., Chuan, Xiong., Xin, Jin., Ping, Li., Xu, Wang., Qiang, Li. 2021. Comparative Mitogenomic Analysis Reveals Dynamics of Intron Within and Between Tricholoma Species and Phylogeny of Basidiomycota., Front. Genet. 12: 534871.

Wenbo, Shi., Weicai, Song., Yuan, Peng., Shuo, Wang., Guiwen, Yang., Chao, Shi. 2022. The complete mitochondrial genome sequence and annotation of Tylopilus plumbeoviolaceoides TH Li, B. Song YH Shen, 2002 (Boletaceae, Boletoideae), Mitochondrial DNA B, 7.6: 999-1000.

Xinrui, Liu., Xiaoping, Wu., Hao, Tan., Baogui, Xie., Youjin, Deng. 2020. Large Inverted Repeats Identified By Intra-Specific Comparison Of Mitochondrial Genomes Provide Insights Into The Evolution Of *Agrocybe Aegerita*, Comput. Struct. Biotec. 18: 2424-2437.

Xu, Wang., Yajie, Wang., Wen, Yao., Jinwen, Shen., Mingyue, Chen., Ming, Gao., Jiening, Ren., Qiang, Li., Na, Liu. 2020. The 256 kb mitochondrial genome of *Clavaria fumosa* is the largest among phylum Basidiomycota and is rich in introns and intronic ORFs., IMA Fungus, 11.1: 26.

Yuting, Zheng., Liangliang, Chen., Kuan, Zhao. 2023. Complete mitochondrial genome sequence of *Lanmaoa* macrocarpa (Boletales, Basidiomycota), Mitochondrial DNA B, 8.10: 1067-1070.

Yasumasa, M., Masaya, N., Katsuhiko, B. 2005. Molecular cloning of developmentally specific genes by representational difference analysis during the fruiting body formation in the basidiomycete *Lentinula edodes*., Fungal. Genet. Biol. 42.6: 493-505.
